# Jianpi Yangzheng Xiaozheng decoction alleviates gastric cancer progression via suppressing exosomal PD-L1

**DOI:** 10.3389/fphar.2023.1159829

**Published:** 2023-08-03

**Authors:** Yanzhen Chen, Jiayun Liu, Yuxuan Chen, Ruijuan Zhang, Jialei Tao, Xu Chen, Haidan Wang, Qingmin Sun, Jian Wu, Shenlin Liu

**Affiliations:** ^1^ Jiangsu Province Hospital of Chinese Medicine, Affiliated Hospital of Nanjing University of Chinese Medicine, Nanjing, Jiangsu, China; ^2^ No. 1 Clinical Medical College, Nanjing University of Chinese Medicine, Nanjing, Jiangsu, China

**Keywords:** traditional Chinese medicine, PD-L1, exosomes, gastric cancer, myeloid-derived suppressor cells, tumor microenvironment

## Abstract

Jianpi Yangzheng Xiaozheng decoction (JPYZXZ) is an empirical traditional Chinese medicine formula that has been reported to significantly prolong the survival of patients with advanced gastric cancer (GC). However, its underlying mechanism have not been fully elucidated. The present work aims to explore the possible mechanism of JPYZXZ on regulating GC progression. We firstly confirmed the inhibitory effect of JPYZXZ in GC MKN74 cells and 615-strain mice, which was possibly mediated with IL-6/JAK2/STAT3 pathway dependent PD-L1 expression. Moreover, we showed that JPYZXZ diminished the expression levels of GC-derived exosomal PD-L1 in MFC murine cells and xenograft GC model, as well as stage IIA-IIIB GC patients. We further found that in different types of tumor-infiltrating immune cells, PD-L1 expression was most positively correlated with myeloid-derived suppressor cells (MDSCs) in GC in the TISIDB database. We isolated exosomes derived from supernatants of MFC cells and co-cultured with bone marrow cells derived from C57BL/6 mice, and further revealed that the expansion of MDSCs was mediated by GC-derived exosomal PD-L1. Meanwhile, our results indicated that JPYZXZ inhibited the delivery of exosomal PD-L1 from GC cells to bone marrow cells, thereby alleviating exosomal PD-L1-induced differentiation and expansion of MDSCs in the tumor microenvironment. This led to a decrease in the levels of several immunosuppressive factors, including iNOS, Arg-1, TGF-β, IL-10, and IL-6, in 615-strain mice. Moreover, clinical data also revealed a significant positive relationship between exosomal PD-L1 and polymorphonuclear MDSCs under the JPYZXZ treatment in stage IIA-IIIB GC patients. In conclusion, our study confirmed that exosomal PD-L1 could be a key factor in controlling MDSCs differentiation in GC. JPYZXZ alleviated GC progression via suppressing exosomal PD-L1 mediated expansion of MDSCs, thereby remodeling the immunosuppressive tumor microenvironment, which provided the experimental evidence for the clinical application of JPYZXZ in the treatment of GC via PD-L1.

## 1 Introduction

Gastric cancer (GC) is one of the most common malignancies of the digestive system. Despite the multidisciplinary treatment options available for patients with advanced GC, the prognosis remains poor. Immunotherapy has been increasingly used in the treatment of solid tumors; by blocking immune checkpoints, anti-programmed cell death ligand 1 (PD-L1) therapy aims to disrupt immune tolerance mechanisms, reactivate T cells, and boost anti-tumor immunity. However, a significant proportion of cancer patients (>70%) do not respond to immune checkpoint inhibitors (ICBs). In GC, several clinical trials have shown a low response rate of approximately 11.6% for treatments targeting PD-L1 (Fuchs et al., 2018). An immunosuppressive tumor microenvironment (TME) represents a major barrier for the responsiveness to PD-L1 inhibitors and other ICBs (Lesterhuis et al., 2017; Alemohammad et al., 2022). Myeloid-derived suppressor cells (MDSCs) are immature, heterogeneous cells that suppress T-cell function and lymphocytes activation by forming an immunosuppressive TME (Cabrera and Marcipar, 2019). Meanwhile, the recruitment of MDSCs initiates the pre-metastatic niches, greatly increasing the likelihood of metastasis (Wang et al., 2019). The accumulation of MDSCs in GC patients is closely correlated with the staging and poor prognosis (Choi et al., 2016). Moreover, the limited response rate of GC immunotherapy is positively related to the high frequency of MDSCs (Koh et al., 2021). Based on the role of MDSCs, targeting MDSCs may be a new strategy for anti-GC.

Exosomes are components of the endocytic pathway that are 40–150 nm in diameter and contain a variety of bioactive molecules. Tumor cells secrete exosomes to modulate immune cells and induce a tumor-supporting microenvironment (Tian et al., 2019). Further research showed that exosomal PD-L1 may account for the ineffectiveness of ICBs (Chen et al., 2018). Compared with soluble PD-L1 or other forms of PD-L1, exosomal PD-L1 are more stable and have stronger immunosuppressive effect. Studies have confirmed that exosomal PD-L1 are easier to be phagocytosed by tumor-associated macrophages (TAMs), platelets, endothelial cells and could exacerbate the immunosuppressive microenvironment (Chen et al., 2021; Pang et al., 2021). The levels of exosomal PD-L1 have been shown to predict the survival and immune status of patients with GC (Fan et al., 2019). However, few studies have been devoted to investigate the potential regulatory role of exosomal PD-L1 and MDSCs, especially in GC.

Traditional Chinese medicine (TCM) has gradually been recognized one of the most important alternative and complementary treatment option, with tremendous therapeutic potential in the prevention and treatment of GC (Wang et al., 2021). Under the guidance of clinical practice and TCM theory, Prof. Shen-Lin Liu demonstrated that the occurrence and development of GC is often caused by lack of righteousness and immune dysregulation. Prof. Liu has used Jianpi Yangzheng Xiaozheng decoction (JPYZXZ) as an adjuvant treatment for patients with GC after chemotherapy, which significantly prolong the survival time and reduced the side effect from chemotherapy (Pan et al., 2020), and the components of JPYZXZ also inhibit the progression of GC *in vivo* and *in vitro* (Wu et al., 2019). Our previous studies have shown that an herbal compound prescription containing the main components of JPYZXZ, such as *Codonopsis pilosula* (Franch.) Nannf. (Dang-Shen), *Astragalus mongholicus* Bunge (Huang-Qi), *Atractylodes macrocephala* Koidz. (Bai-Zhu), *Angelica sinensis*(Oliv.)Diels (Dang-Gui), *Sparganium stoloniferum* Buch.-Ham (San-Leng) and *Curcuma zedoria* (Christm.) Roscoe (E-zhu), *Citrus reticulata* Blanco (Chen-Pi) and *Glycyrrhiza uralensis* Fisch. (Gan-Cao), can exert anti-cancer effect by reducing the expression of PD-L1 in GC (Xu et al., 2020). Furthermore, our recent study also found that modified JPYZXZ inhibit tumor growth and metastasis by regulating GC exosomes (Wu et al., 2022). However, whether the JPYZXZ has a regulatory effect on exosomal PD-L1 in GC have not been systematically reported.

In this study, we evaluated the inhibitory effect of JPYZXZ on PD-L1 in GC MKN74 cells. We systematically investigated the effect of JPYZXZ on GC-derived exosomal PD-L1 in MFC cells, xenograft tumor mouse model and advanced GC patients. We specifically focused on the potential regulatory effect of exosomal PD-L1 on the expansion of MDSCs in the TME, and the underlying the effects of JPYZXZ (Supplementary Figure S1). Based on our experiment, we confirm the importance of exosomal PD-L1 for the treatment of GC, and provide the novel evidence in support of the clinical application of JPYZXZ in treating GC patients.

## 2 Materials and methods

### 2.1 Composition and preparation of JPYZXZ decoction

The botanical drugs of JPYZXZ was prepared using *A. mongholicus* Bunge (Huang-Qi); *C. pilosula* (Franch.) Nannf. (Dang-Shen); *A. macrocephala* Koidz. (Bai-Zhu); *A. sinensis*(Oliv.)Diels (Dang-Gui); *Paeonia lactiflora* Pall. (Bai-Shao); *S. stoloniferum* Buch.-Ham (San-Leng); *C. zedoria* (Christm.) Roscoe (E-Zhu); *Vladimiria souliei* (Franch.) Ling (Mu-Xiang); *C. reticulata* Blanco (Chen-Pi); *Scleromitrion diffusum* (Willd.) R.J.Wang (Bai-Hua-She-She-Cao); *Salvia chinensis* Benth. (Shi-Jian-Chuan); *G. uralensis* Fisch. (Gan-Cao). The herbal information are shown in Supplementary Table S1. All botanical drugs were purchased from the pharmacy of the Affiliated Hospital of the Nanjing University of Chinese Medicine. Briefly, JPYZXZ decoction is prepared by the following process: the botanical drugs were pooled together and soaked in 1.6 L of distilled water for half an hour, brought to a boil over high heat, and continued to decoct for 1 h. After filtration of the liquid, the drug residue were boiled again with 1.6 L added distilled water for 40 min. The two filtrates were mixed together, filtrated and concentrated after cooling. The concentrated solution (with a relative density of 1.16) was subjected to alcohol precipitation by adding 95% ethanol to attain an alcohol content of 85%. Following a 24 h period of settling at room temperature, the mixture was filtered and subsequently freeze-dried. Approximately 7.3 g of extract could be obtained from 203 g of raw herbs. For cellular experiments, the extract was filtrated with a 0.22 μm filter for use in accordance with the experimental requirements.

Orthogonal experimental design and conventional extraction techniques were used to create 10 batches of JPYZXZ for UPLC–Orbitrap–MS analysis. Based on the above-mentioned traditional decoction method, the two filtrates of botanical drugs were filtrated, concentrated, alcohol precipitated, freeze-dried to powder and marked as S10. The effect of four influencing factors (solid-liquid ratio, soaking time, boiling time, and decoction times) were used, and each of which was set at three levels to create an orthogonal table. According to the conditions listed in Supplementary Table S2, nine batches of JPYZXZ samples were boiled and labeled with S1–S9. Each freeze-dried powder was weighed precisely and dissolved in 100 μL 20% methanol (50 mg/mL) for UPLC–Orbitrap–MS analysis.

### 2.2 UPLC–Orbitrap–MS conditions

The sample was analyzed using an Ultimate 3000UPLC system (Dionex, United States) combined with a BEH C8 column (2.1 × 100 mm, 1.7 µm) (Waters, United States) and a HSS T3 column (2.1 × 100 mm, 1.8 µm) (Waters, United States). The temperature of the column was kept at 30°C, the flow rate was 0.35 mL/min, and the sample injection volume was 5 μL. In the positive ion mode, the mobile phase was consisted of water with 0.1% formic acid (solvent A) and 0.1% formic acid in acetonitrile (solvent B). The gradient profile was performed as follows: 5% B (0–1 min); 5%–100% B (1–24 min); 100% B (24.1–27.5 min); 5% B (7.6–30 min). In the negative ion mode, the mobile phase was a mixture of water with 6.5 mM ammonium bicarbonate (solvent A) and 95% methanol with 6.5 mM ammonium bicarbonate (solvent B). Gradient elution condition: 5% B (0–1 min); 5% B (1–18 min); 100% B (18.1–22 min); 5% B (22.1–25 min). The electrospray ionization (ESI) source was operated and optimized and the MS parameters were as follows: aux gas flow rate: 8 Arb; sheath gas flow rate: 35 Arb; capillary temperature: 320°C; aux gas heater temperature: 350°C; mass range: 70–1,050 m/z, the full MS resolution: 70000; MS/MS resolution: 17500. The collision energy was 20, 40% in NCE mode; the spray voltage was +3.8 kV (positive) or −3.0 kV (negative).

### 2.3 Fingerprint established and evaluated

For method validation, the precision, stability, and repeatability were examined. Six consecutive injections of the same sample solution were used to calculate precision, and six samples from the same source were used to assess repeatability. The sample stability test was evaluated by injecting the same sample solution over the course of 1 day (0, 4, 8, 12, 16, and 24 h). Quality control (QC) samples were prepared by pooling the same amount of JPYZXZ from ten samples together (500 mg/mL). Tryptophan-d5 was used as internal standard (IS). To determine the relative retention time (RRT) and relative peak area (RPA) of each common peaks, the IS peak was chosen as the reference. For individual metabolites in the 10 batches of JPYZXZ, the coefficients of variation (CVs %) of RRT and RPA were examined. Using the TCM chromatography fingerprint system (Version 2012A) for similarity evaluation.

### 2.4 Cell lines and cell culture

Human MKN-74 and MFC murine GC cell lines were purchased from the Cell Bank of the Chinese Academy of Sciences (Shanghai, China). The cells were cultured in RPMI 1640 medium (Gibco, Grand Island, United States) supplemented with 10% newborn calf serum (Evergreen Company, China) at 37°C and 5% CO_2_. In all experiments, cells were cultured to 80%–90% confluence. The medium was changed every 24–48 h.

### 2.5 Cell viability assay

The IC50 of the JPYZXZ intervention MKN74 in the CCK8 experiment was calculated in cellular experiments to determine the compound dose. A dose range of 0, 2, 4, 8, 16, 32, 64, 128, and 256 mg/mL was selected for test, and approximately 50% of the cells were inhibited after 24 h of exposure. Briefly, cells were seeded into 96-well plates at a cell concentration of 5 × 10^3^ cells/well, treated with different concentrations of JPYZXZ, and then cultured at 37°C for 24 h. CCK8 (Dajinbo, KV500) solution was added to each well and incubated for 1 h at 37°C. The absorbance of each well was measured at 450 nm. The IC50 values were calculated using GraphPad Prism 8 (San Diego, CA, United States).

### 2.6 Isolation and identification of exosomes from serum and cell culture supernatants

Serum sample with volume of 400 µL was firstly centrifuged at 10,000 g centrifuge at 4°C for 30 min to remove cells and fragments. The sample was added 0.25 volume of ExoQuick (System Biosciences, EXOQ5TM-1) reagent according to the manufacturer’s instructions. After incubation for 16 h at 4°C, the mixture of serum and reagent were centrifuged at 1,500 g for 30 min to pellet exosomes. Finally, the exosomal pellet was resuspended in 100 μL of PBS and stored in refrigerator at −80°C for the following experiments.

Exosomes isolated from the cell culture medium were sequentially centrifuged at 800 g at 4°C for 5 min, 2,000 g at 4°C for 10 min, 10,000 g at 4°C for 30 min, and ultracentrifuged at 100,000 g at 4°C for 80 min. After discarding the supernatant, the pellet was resuspended in PBS and secondly ultracentrifuged at 100,000 g at 4°C for 80 min. The final pellet containing the exosomes was resuspended in PBS and then stored at 4°C for short term (1–7 days) or −80°C for long term storage. Subsequently, the morphology of exosomes were identified using transmission electron microscope. Firstly, 20 μl of an exosome suspension was placed on a copper mesh with liquid removed for 3 min, and counterstained with 2% phosphotungstic acid solution for 10 min. After being dried for 2 min under incandescent light, the exosomes were photographed under a transmission electron microscopy (TEM, Tecnai-12; Philips, Netherlands). The expression of the exosomal markers CD9, CD63 and TSG101 was examined by Western blot analysis. Particle size and concentration were performed using nanoparticle tracking analysis (NTA, Particle Metrix, Germany).

### 2.7 Lentivirus generation and transfection

The lentivirus generation protocol was primarily based on previous methods (Nasri et al., 2014). Briefly, PD-L1 sequence from mouse full-length cDNA was amplified by Shanghai GeneChem and subcloned into the GV492 vector. The vector elements were Ubi-MCS-3FLAG-CBh-gcGFP-IRES-puromycin. Using lipo3000 (Thermo), the vector were co-transfected into the 293T cells in accordance with the manufacturer’s protocol. After 3 days, the PD-L1 over-expressing lentivirus was harvested and stored at −80°C. Due to demonstration of adequate upregulation of PD-L1 gene expression by inverted fluorescence microscope observation and Western blotting, the dilution was set at a concentration of 20 MOI for subsequent experiments.

### 2.8 Western blot assay

Total protein samples were extracted using RIPA lysis buffer. A nucleic acid and protein microanalyzer (Molecular Devices, United States) was used to determine protein concentrations. Western blotting was performed according to a published protocol (Wu et al., 2022). Primary antibodies were incubated overnight at 4°C as follows: anti-iNOS (Proteintech, 22226-1-AP, 1:5000), anti-PD-L1 (Proteintech, 66248-1-Ig, 1:3000), anti-Arg-1 (Proteintech, 16001-1-AP, 1:2000), anti-GADPH (Proteintech, 60004-1-Ig, 1:10000), anti-CD9 (Proteintech, 20597-1-AP, 1:2000), anti-CD63 (Proteintech, 25682-1-AP, 1:2000), anti-TSG101 (Proteintech, 28283-1-AP, 1:3000), anti-JAK2 (Cell Signaling Technology, 3230S, 1:2000), anti-STAT3 (Proteintech, 10253-2-AP, 1:5000), anti-p-STAT3 (Cell Signaling Technology, 9145S, 1:1000), and anti-IL-6 (Cell Signaling Technology, 12912S, 1:1000). The HRP-conjugated secondary antibody (ZSGB-BIO, ZB-2305; ZB-2301) was incubated for 1 h at room temperature. The signal was detected using the ECL method (Beyotime, China, P0018FS). Band density was quantified using ImageJ software (NIH, Bethesda, MD, United States) for the grayscale intensity of each protein.

### 2.9 Preparation of GW4869 solution

We used a standard exosome secretion inhibitor, GW4869 (MedChemExpress, HY-19363) using a standard protocol. First of all, we centrifuged the product at low speed to obtain the GW4869 powder. Subsequently, 5 mg of GW4869 was dissolved 432.9 μL of DMSO (0.005%) at a concentration of 20 mM as previously described. Then, the solution was diluted by 0.9% normal saline, and the injection dose was 2.5 μg/g body weight (Essandoh et al., 2015; Wang et al., 2019).

### 2.10 Establishment of xenograft tumor model

Mice from the 615 line were purchased from the Tianjin Institute of Blood (male, 6–8 weeks old, weighing 20 ± 1 g). Each mouse was subcutaneously inoculated with 200 μL of cell suspension (5 ×10^7^/mL, MFC murine GC cells) in the right axilla. After the tumor volume reached 50–100 mm^3^, the mice were randomly divided into four groups: model group (model), JPYZXZ-low dose group (JPYZXZ-L), JPYZXZ-high dose group (JPYZXZ-H), and exosome secretion inhibitor hydrochloride hydrate (GW4869) group (GW4869 2.5 μg/g via intraperitoneal injection). The low, high dose JPYZXZ gavage groups were each treated with 470 mg/kg, 950 mg/kg of extract (equivalent to JPYZXZ raw herbs 13.20 g/kg, 26.4 g/kg, respectively). The optimal JPYZXZ dose level was calculated using the clinical dose of JPYZXZ and a dose-equivalence factor based on surface area between species (Nair and Jacob, 2016). Mice were weighed and the tumor volumes were measured once every 2 days according to the formula (0.523 × length × width^2^). On day 21, the mice were sacrificed by an overdose of pentobarbital sodium (100 mg/kg i.p.), and blood and tumor tissues were collected. Animal experiments were performed in accordance with the animal ethical guidelines and approved by the Experimental Animal Ethics Committee of Nanjing University of Traditional Chinese Medicine (No. 2021DW-35-01).

### 2.11 MDSCs were generated from bone marrow (BM) progenitors

BM cells were isolated as described previously (Zhang et al., 2020). Briefly, BM cells from the femoral and tibial bones were obtained from male C57BL/6 mice (6 weeks old, weighing 18–22 g, purchased from the Tianjin Institute of Blood). Cell suspensions were filtered through a 70-μm cell strainer. The isolated cells were washed with Hank’s buffered salt solution (HBSS) (Gibco, 14065056) and centrifuged at 300 g for 6 min at 4°C, resuspended in erythrocyte lysis buffer (Beyotime, C3702) for 5 min at room temperature. Following this, the BM cell suspension was diluted to 5 × 10^5^ cells/mL and inoculated into 6-well plates. Based on previous studies *in vitro*, the concentration of exosomes was 50–200 μg/mL (Sahin et al., 2019; Zhang et al., 2020), therefore, we choose 100 μg/mL exosomes for the subsequent induction. BM cells were cultured in RPMI 1640 medium supplemented with 10% exosome-depleted FBS, 20 ng/mL granulocyte and macrophage colony-stimulating factor (GM-CSF) (Peprotech, 031955), with or without exosomes extracted from supernatants of MFC cells (NC-exo, 100 μg/mL), and exosomes from MFC cells overexpressing PD-L1 (PD-L1-OE-exo, 100 μg/mL). After incubation for 4 days, cell phenotypes were determined using flow cytometry.

### 2.12 Immunofluorescence staining and hematoxylin and eosin (H&E) staining

Dil dye (Beyotime, C1036) was added to 50 μL of exosome solution and then incubated at 37°C for 30 min. Dil-labeled exosome solution (1 μg/μL) was added to BM-derived MDSCs, followed by incubation at 37°C for 48 h with or without JPYZXZ. The culture medium was discarded, and fixed samples were blocked with blocking buffer (Beyotime, P0260) for 1 h, washed twice with PBS, and then incubated with anti-PD-L1 (Proteintech, 66248-1-Ig, 1:200) overnight. Next, the sections were incubated with fluorescent secondary antibodies and DAPI for 10 min. In addition, we analyzed tumor tissue and lung tissue sections from mice using the primary antibodies against CD11b (Abcam, ab13357, 1:100), anti-Gr-1 (BioLegend, 108448, 1:100), and anti-PD-L1 (Proteintech, 66248-1-Ig, 1:200). The sections were examined using fluorescence microscope and measured using ImageJ. Moreover, tumor tissues were fixed in 4% paraformaldehyde and embedded in paraffin, and then stained with H&E.

### 2.13 Flow cytometry analysis

Tumor tissues were isolated and cleaned in ice-cold PBS before the tissue was cut into 0.1 mm^3^ pieces. The cells were isolated by incubating the tissues in digestion buffer, including RPMI 1640, 5% FBS, 1 mg/mL collagenase IV (BioFroxx, 2091MG100), 0.2 mg/mL DNaseI (Sigma-Aldrich, D2821), and 0.2 mg/mL hyaluronidase (BioFroxx, 1141MG100). Suspensions of tumor pieces were incubated at 37°C for 1 h with shaking. The digested tissues were filtered through 70 μm cell strainers and the cells were collected by centrifugation at 800 g for 5 min at 4°C. The single-cell suspensions (1 × 10^7^ cells/mL) were stained with fluorochrome-coupled antibodies AF488-CD45 (Elabscience, E-AB-F1136L), PE/Dazzle™594-CD11b (BioLegend, 101256), and APC-Gr-1 (BioLegend, 108412) for 30 min at 4°C. Cells were analyzed using FACSVerse (BD Biosciences, MA).

The blood of tumor-bearing mice was prepared on day 10, and approximately 200 μL per mouse was harvested. The erythrocytes of blood cells were then removed by erythrocyte lysis buffer (Beyotime, C3702) for 10 min at room temperature, and the lysis was terminated with PBS followed by centrifugation at 300 g for 4 min. The stained cells were incubated with the same antibodies as the tissues. Human MDSCs in peripheral blood were stained with fluorochrome-coupled antibodies PE-CY7-CD11b (BD Pharmingen, 557743), PE-CD14 (BioLegend, 301806), FITC-CD15 (BioLegend, 323004), and APC-HLA-DR (BioLegend, 307610). After the washing step, the cells were analyzed using a FACSVerse (BD Biosciences, MA). The results were analyzed using the FlowJo software.

### 2.14 Elisa procedures

Exosomes pellets isolated from 200 μL serum were resuspended using exosome binding buffer for ELISA. The total protein of exosomes was determined using the BCA Protein analysis kit (Beyotime Biotechnology, Shanghai, China). A human PD-L1 ELISA kit (JiyinmeiBio, JYM1968Hu) and mouse PD-L1 ELISA kit (JiyinmeiBio, JYM0144Mo) were used for quantitation of the exosomal PD-L1 concentration based on the recommendatory procedures.

IL-6, IFN-γ, IL-10, TNF-α, and TGF-β levels in the peripheral blood of the mice were detected using an ELISA kit (JiyinmeiBio, China, Lot: JYM0012Mo, JYM0540Mo, JYM1123Mo, JYM0005Mo, JYM0218Mo) following the manufacturer’s instructions. The absorbance was measured at a wavelength of 490 nm using a microplate reader.

### 2.15 Patients and clinical data

From May 2019 to August 2022, a total of 30 patients with GC were recruited for this study. The inclusion criteria included histopathological diagnosis of stage IIA-IIIB GC with syndrome of spleen qi deficiency, possess an indication for chemotherapy and willingness to receive chemotherapy. All enrolled patients underwent FOLFIRI chemotherapy and no other treatment. Using the JPYZXZ prescription as an exposure factor, 15 patients were treated with JPYZXZ prescriptions during two cycles of chemotherapy (treatment group), and 15 patients were treated with chemotherapy only (control group). There were 22 males and 8 females, aged 37–74 years. One dose of JPYZXZ was 200 mL, and this was administered twice per day, four times a week for 3 months. Before and after the treatment, peripheral blood was collected from the enrolled patients using EDTA anticoagulation tubes, of which 400 µL was used for MDSCs flow cytometry analysis and 1 mL was centrifuged to isolate serum for exosome extraction. All patients signed an informed consent form, and this study was performed in accordance with the Helsinki Declaration. This clinical trial was approved by the Ethics Committee of the Affiliated Hospital of Nanjing University of Chinese Medicine in compliance with the Helsinki Declaration (No. 2019NL-090-02).

### 2.16 Statistical analysis

Statistical analyses were performed using GraphPad Prism software (version 8.0; San Diego, CA, United States) using Student’s *t*-test (unpaired, two-tailed) or one-way analysis of variance (ANOVA) (followed by Tukey’s *post hoc* tests or least significant differences). Statistical significance was set at *p* < 0.05.

## 3 Results

### 3.1 UPLC–Orbitrap–MS fingerprints of JPYZXZ decoction

#### 3.1.1 Method validation for fingerprint

The precision and reproducibility relative standard deviations (RSD) were both less than 2.80%, and samples for UPLC–Orbitrap–MS analysis remained stable for 24 h at room temperature.

#### 3.1.2 Fingerprint similarity

The similarity between the fingerprint of each sample of different samples and the reference fingerprint ranges from 0.964 to 0.992 in the positive ion mode. The similarity of S4 was 0.964, and all other samples were more than 0.967. In the negative ion mode, the similarities between the reference fingerprint and the S1–S10 spectra ranged from 0.881 to 0.995. The similarity of S5 was 0.881, and the other batches were more than 0.974. The detailed results of similarity evaluation were shown in Supplementary Table S3.

#### 3.1.3 UPLC–Orbitrap–MS fingerprint

**FIGURE 1:**
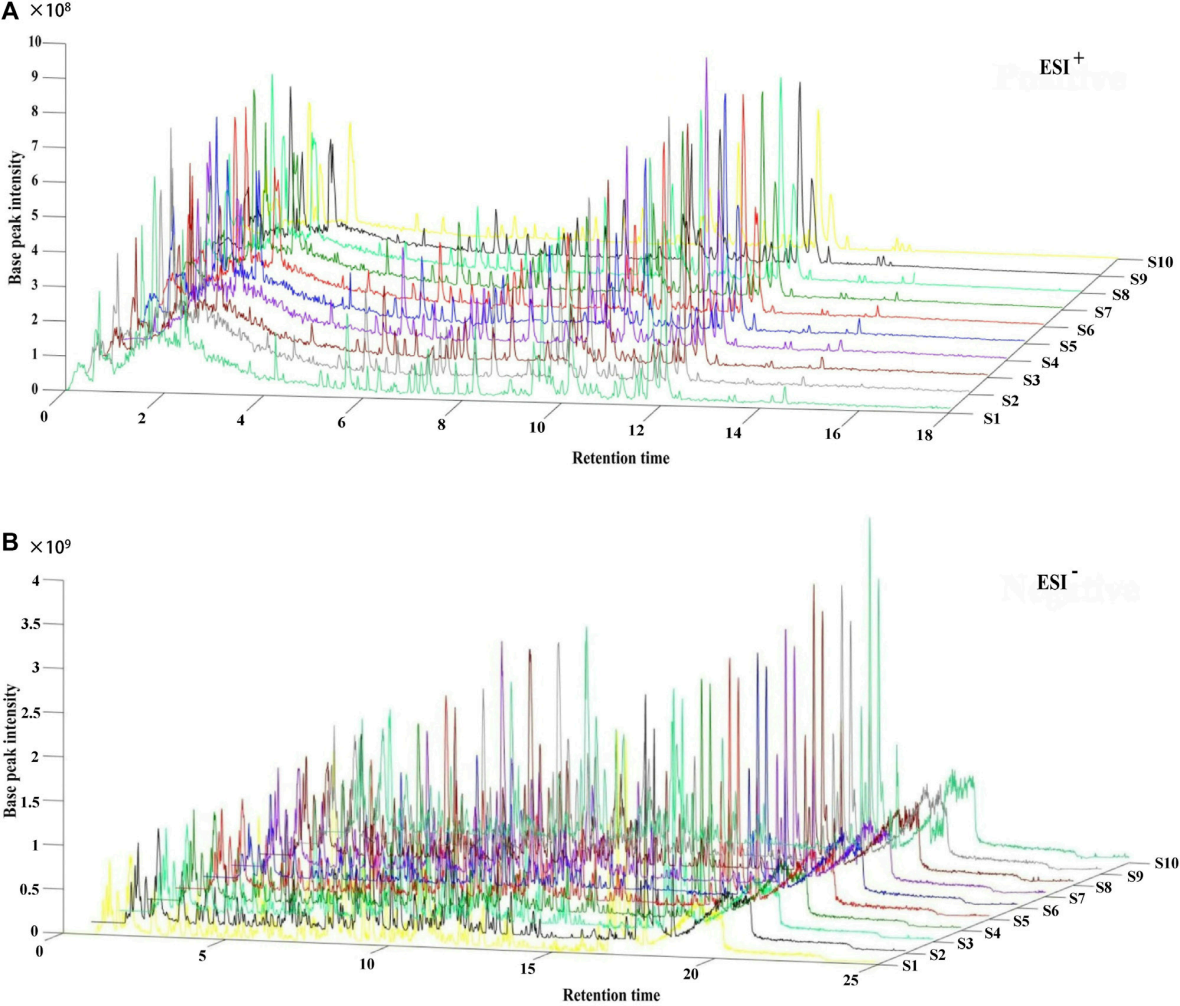
UPLC–Orbitrap–MS fingerprints of ten batches of JPYZXZ. **(A)** positive ion mode; **(B)** negative ion mode.

**FIGURE 2:**
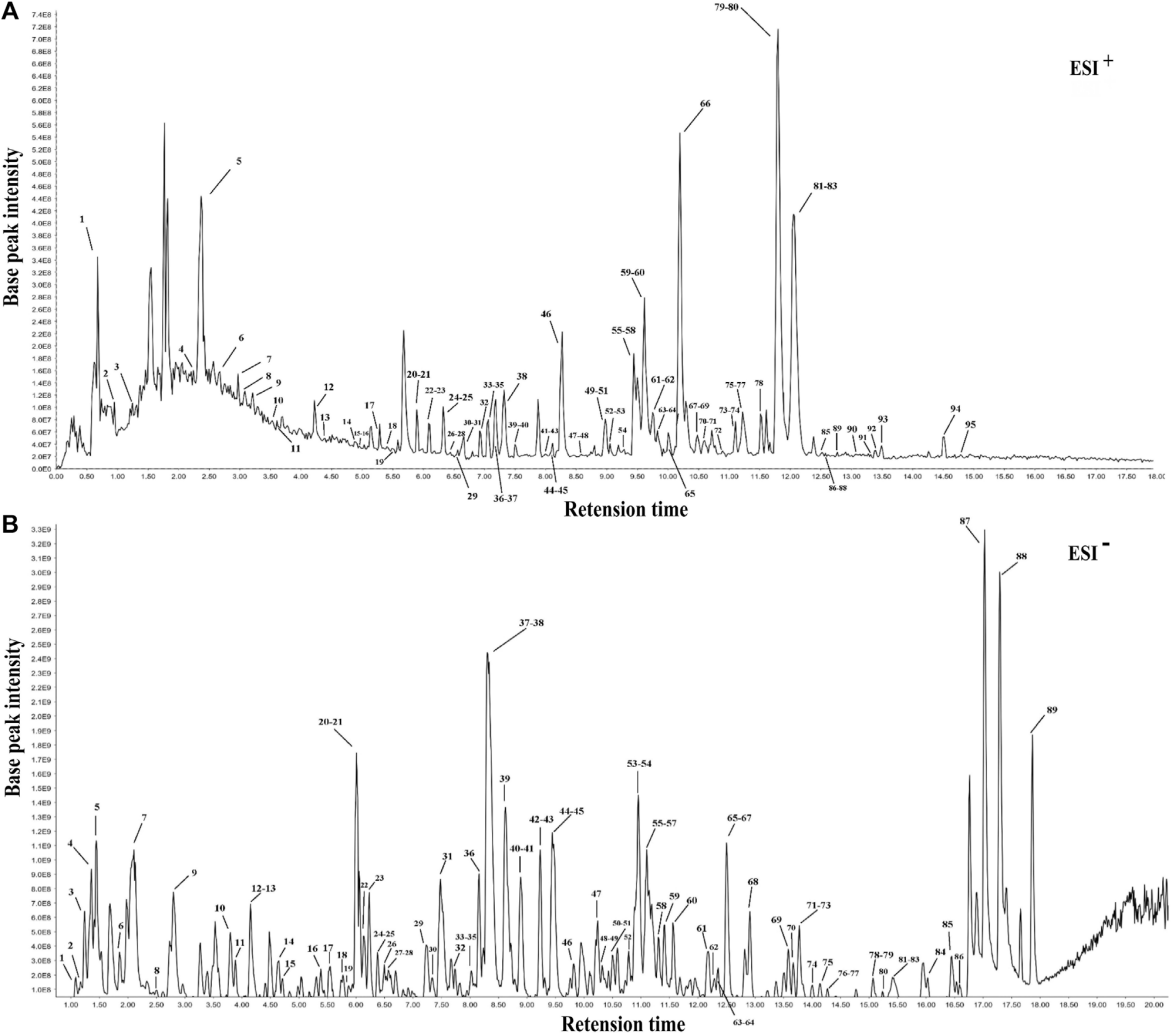
The reference fingerprints of JPYZXZ. **(A)** positive ion mode; **(B)** negative ion mode.

Following the similarity analysis of the profiles of ten batches of JPYZXZ, UPLC–Orbitrap–MS fingerprints (Figure 1) were generated. The reference fingerprints were shown in Figure 2. In the positive and negative ion modes, the CV% of RRT were 0.03%–1.81% and 0.02%–1.69%, respectively. Meanwhile, the coefficients of variation of RPA were respectively 2.99%–147.97% and 1.84%–62.41%. The results were included in Supplementary Tables S4–S7. After removing the duplicates, a total of 177 common peaks were identified. Detailed information on the metabolites of JPYZXZ, such as retention time, chemical formula and name were shown in Supplementary Tables S8, S9.

### 3.2 JPYZXZ diminished the expression of PD-L1 in GC cells via the IL-6/JAK2/STAT3 axis

**FIGURE 3:**
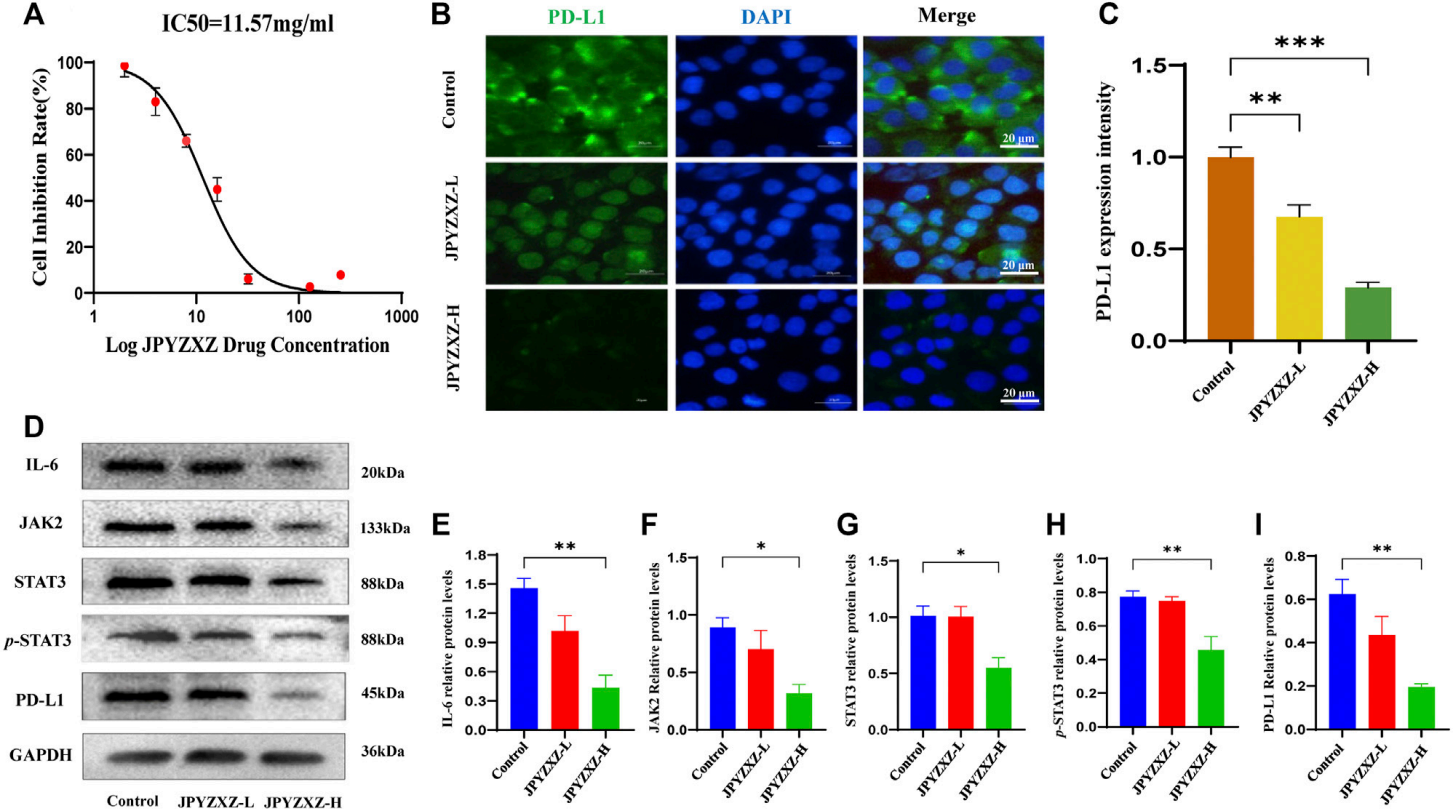
JPYZXZ diminished the expression of PD-L1 in GC cells. **(A)** The drug dosage curve of JPYZXZ on gastric cells after 24 h of exposure. **(B,C)** Representative images of PD-L1 immunofluorescence in MKN-74 cells. Scale bar = 20 μm. **(D–I)** Western blot analysis of PD-L1 and proteins in the IL-6/JAK2/STAT3 signaling pathway after JPYZXZ treatment. Data are expressed as mean ± SEM, *n* = 3 independent experiments. *p < 0.05, **p < 0.01.

Previous studies showed that the expression of PD-L1 in MKN74 cells (Fan et al., 2019) is significantly higher than in other GC cells. We tested MKN74 cells using the CCK8 assay and founded that approximately 50% of the cells were inhibited after 24 h of exposure to 11.57 mg/mL of JPYZXZ (Figure 3A). Based on the IC50, we chose 12 and 6 mg/mL as the high and low doses for the follow-up experiments.

Available research indicated that IL-6/JAK/STAT3 signal could regulate PD-L1 expression (Zhang et al., 2016; Chan et al., 2019). Accordingly, the following immunofluorescence staining showed a decrease in the expression of PD-L1 in the JPYZXZ-L (*p* < 0.01) and JPYZXZ-H (*p* < 0.001) compared with that in control group (Figures 3B, C). Western blot showed that the JPYZXZ-H decreased protein expression of JAK2 (*p* < 0.05), STAT3 (*p* < 0.05), IL-6 (*p* < 0.01) and *p*-STAT3 (*p* < 0.01). Furthermore, JPYZXZ decreased the PD-L1 expression levels in GC cells, with more pronounced effects in the JPYZXZ-H (*p* < 0.01) compared with the control group (Figures 3D–I). Therefore, we assumed that JPYZXZ possibly regulated the IL-6/JAK2/STAT3 pathway, which contributes to the inhibitory effect of JPYZXZ on PD-L1.

### 3.3 JPYZXZ decreased comtent of exosomal PD-L1 in GC cells and diminished differentiation of MDSCs induced by exosomal PD-L1

**FIGURE 4:**
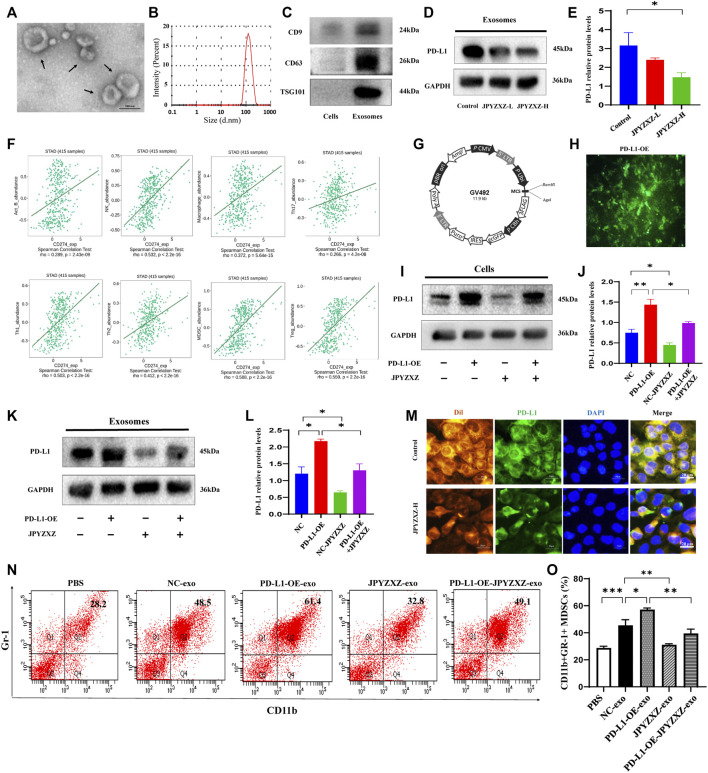
JPYZXZ reversed expansion and differentiation of MDSCs caused by GC cell exosomal PD-L1. **(A)** Transmission electron microscopy image of exosomes. **(B)** NTA analysis confirmed the exosomes. **(C)** Western blot of the exosome-related markers in GC cell-derived exosomes. **(D,E)** Western blot analysis of exosomal PD-L1 in GC cells. **(F)** Correlation of PD-L1 expression with infiltrating levels of MDSCs and immune cells in STAD. **(G,H)** Fluorescence images of PD-L1 after lentivirus transfection of MKN-74. **(I,J)** Western blot analysis of the PD-L1 content in GC cells. **(K,L)** Western blot analysis of the exosomal PD-L1 content in culture supernatant of GC cells. **(M)** Immunofluorescence staining for DAPI (nucleus), Dil (exosomes), and PD-L1 in MDSCs. Scale bar = 20 μm. **(N,O)** Percentages of CD11b+Gr-1+ MDSCs detected using flow cytometry. Data are expressed as mean ± SEM, *n* = 3 independent experiments. *p < 0.05, **p < 0.01, ***p < 0.001.

To determine the effect of JPYZXZ on PD-L1 in GC-derived exosomes, we further extracted exosomes from the supernatant of the GC cells. Exosomes are 40–150 nm extracellular vesicles with double membranes and are positive with CD9, CD63 and TSG101 (Figures 4A–C). Western blot results showed that the level of exosomal PD-L1 decreased in the JPYZXZ-H (*p* < 0.05) when compared to the control group (Figures 4D, E), suggesting the potential regulatory effect of JPYZXZ on GC cells-derived exosomal PD-L1.

To elucidate the effect of the PD-L1 secreted by GC cells on tumor-infiltrating immune cells in the TME, an integrated repository portal for tumor-immune system interactions (TISIDB) web server was used. We evaluated the correlation of PD-L1 and Th1, TH17, Th2, Treg, B cells, macrophages, NK cells and MDSCs in gastric adenocarcinoma (STAD), and found that PD-L1 was most positively correlated with MDSCs in STAD (*R* = 0.588, *p <* 0.0001) (Figure 4F). We then aimed to investigate the role of exosomal PD-L1 in MDSCs differentiation. We firstly established lentivirus transfection of GC cells and validated them using fluorescence microscopy and Western blotting (Figures 4G, H). We further found that PD-L1 in cells and exosomes was significantly increased when PD-L1 was overexpressed (*p* < 0.01, *p* < 0.05), while JPYZXZ treatment could reverse these phenomena (*p* < 0.05, *p* < 0.05), however, PD-L1 overexpression attenuated these inhibitory effect (Figures 4I–L), which supported that exosomal PD-L1 is potential target of JPYZXZ in GC.

There are proportions of BM cells in mice contain the phenotype of MDSCs (Gabrilovich and Nagaraj, 2009). To investigate whether GC exosomal PD-L1 would stimulate MDSCs (CD11b+Gr-1+) expansion, MDSCs were generated from BM progenitors as described previously (Zhang et al., 2020). BM-derived MDSCs were cultured with exosomes derived from supernatants of MFC cells (NC-exo) labelled with red fluorescent Dil. The immunofluorescence indicated efficient uptake of Dil-labeled exosomes and PD-L1 by MDSCs, whereas JPYZXZ could reduce the content of exosomal PD-L1 (Figure 4M). Flow cytometry analysis showed that NC-exo significantly promoted MDSCs expansion compared with the PBS group in our experiment (*p* < 0.001). Notably, we extracted exosomes derived from MFC cells after PD-L1 overexpression and found that PD-L1-OE-exo could dynamically promote expansion of MDSCs compared with the NC-exo group (*p* < 0.05). Subsequently, we evaluated whether JPYZXZ could affect the exosomal PD-L1 induced MDSCs and found that after treatment of JPYZXZ, the expansion of MDSCs in NC-exo (*p* < 0.01) and PD-L1-OE-exo (*p* < 0.01) were significantly diminished, however, PD-L1 overexpression attenuated the inhibitory effect of JPYZXZ in NC-exo (Figures 4N, O). The above results implied that GC-derived exosomes induced the expansion of MDSCs might be mediated through exosomal PD-L1, and JPYZXZ inhibited MDSCs expansion by reducing exosomal PD-L1 expression.

### 3.4 JPYZXZ inhibited tumor growth by reducing levels of the exosomal PD-L1 *in vivo*

**FIGURE 5:**
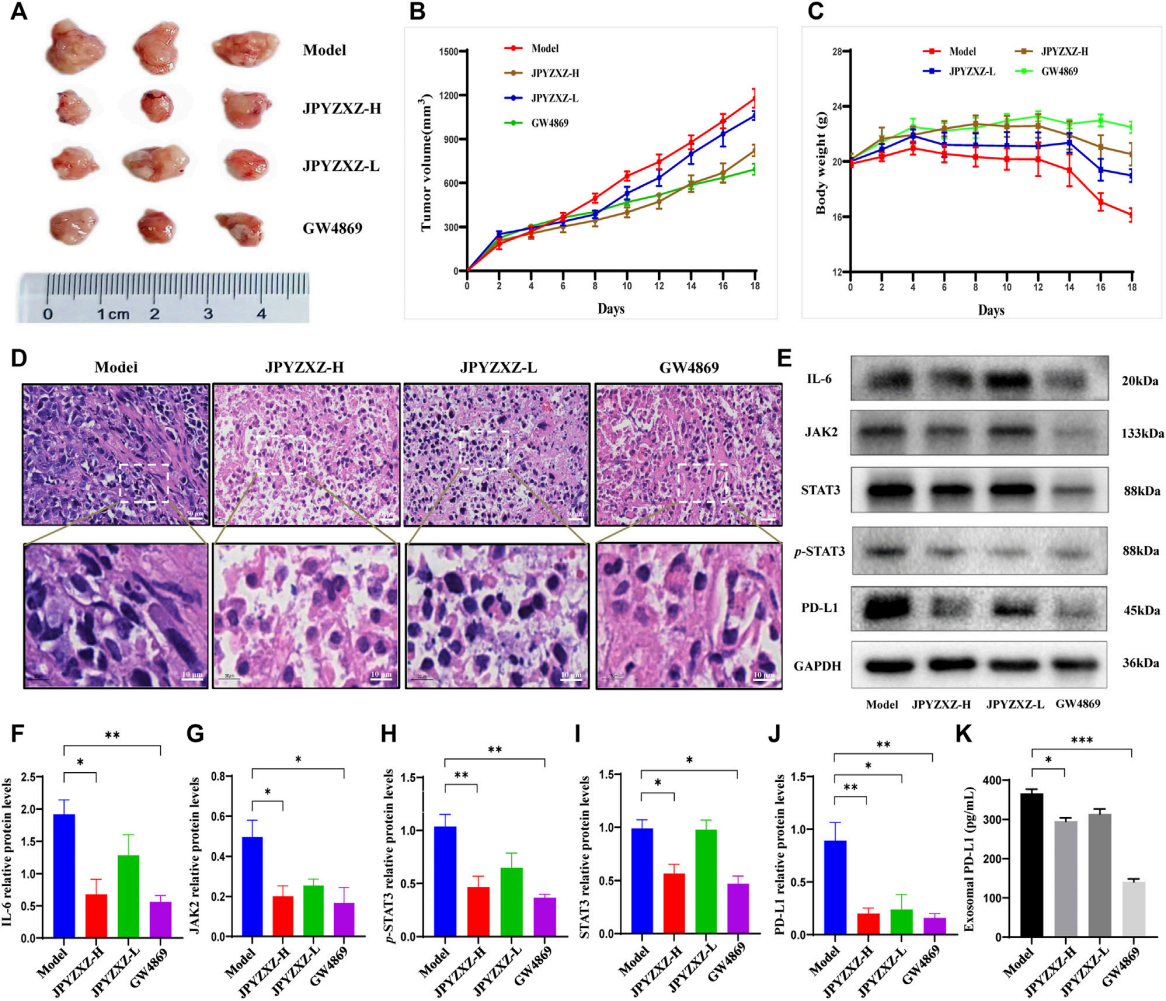
JPYZXZ decreased the expression of PD-L1 and exosomal PD-L1 levels in tumor-bearing mice. **(A)** Representative photographs of tumors. **(B)** Tumor weights on days 2, 4, 6, 8, 10, 12, 14, 16, and 18. **(C)** The body weights were detected every 2 days. **(D)** Representative images of H&E staining of the tumor tissue sections from indicated mice. Upper row: scale bar = 20 μm; lower row: scale bar = 10 μm. **(E–J)** Expression levels of IL-6/JAK2/STAT3 pathway-related proteins and PD-L1 in the tumor tissues. **(K)** Exosomal PD-L1 levels in the serum of tumor-bearing mice. Data are represented as the mean ± SEM, *n* = 5 mice or *n* = 3 independent experiments. *p < 0.05, **p < 0.01, ***p < 0.001.

*In vivo* experiments were next applied to further validate the above-mentioned results. Compared with the model group, tumor volume was significantly reduced with dose dependent in JPYZXZ-treated groups or the GW4869 group (Figures 5A, B), but not body weight (Figure 5C). The H&E staining results indicated that compared with the model group, the cytoplasm of the tumor cells showed obvious eosinophilic changes in the tumor tissues of JPYZXZ-treatment and GW4869 groups, with increased nuclear fragmentation and tumor necrosis (Figure 5D).

Consistent with our *in vitro* experiments, JPYZXZ reduced the expression of IL-6 (*p* < 0.05), JAK2 (*p* < 0.05), STAT3 (*p* < 0.05) and *p*-STAT3 (*p* < 0.01) in the JPYZXZ-H, and the quantitative results also indicated that JPYZXZ-H (*p* < 0.01) group inhibited the expression of PD-L1 better than JPYZXZ-L (*p* < 0.05) compared with the model group (Figures 5E–J). Furthermore, the expression levels of the exosomal PD-L1 were decreased in the serum of tumor-bearing mice in the JPYZXZ-H (*p* < 0.05) in contrast to the model group (Figure 5K). To sum up, these results demonstrated that JPYZXZ inhibited tumorigenesis of 615-strain mice by reducing the expression of PD-L1 in tumor tissues and serum exosomes.

### 3.5 JPYZXZ remodeled the TME by decreasing MDSCs expansion

**FIGURE 6:**
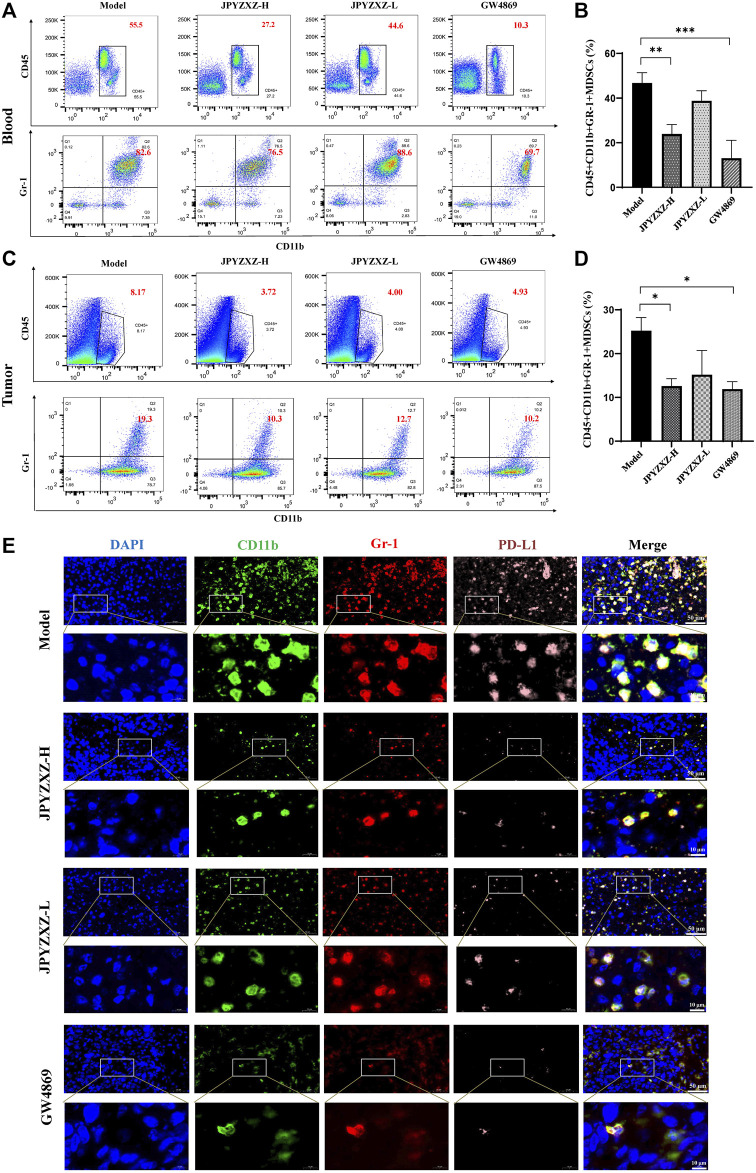
JPYZXZ reduced the number of MDSCs in tumor bearing mice. **(A,B)** Percentage of MDSCs (CD45^+^CD11b+Gr1+) in blood determined using flow cytometry. **(C,D)** Percentage of MDSCs (CD45^+^CD11b+Gr1+) in tumors determined using flow cytometry. **(E)** Immunofluorescence staining for DAPI (nucleus), CD11b, Gr-1, and PD-L1 in tumor tissue sections from the xenograft tumor model. Data are represented as the mean ± SEM, *n* = 5 mice or *n* = 3 independent experiments. *p < 0.05, **p < 0.01, ***p < 0.001.

As shown in Figures 6A, B, quantitative analysis suggested that the proportion of intratumoral MDSCs (CD45^+^CD11b+Gr-1+) was significantly decreased in the JPYZXZ-H (*p* < 0.05) group when compared with the model group. Besides, flow cytometry analysis also showed the frequency of MDSCs was significantly decreased in JPYZXZ-treated groups in blood, especially in JPYZXZ-H (*p* < 0.01) (Figures 6C, D). Simultaneously, immunofluorescence results showed that when compared with the model group, the levels of PD-L1 in the intratumoral content of MDSCs (CD11b and Gr-1) were significantly decreased in a dose dependent manner (Figure 6E).

**FIGURE 7:**
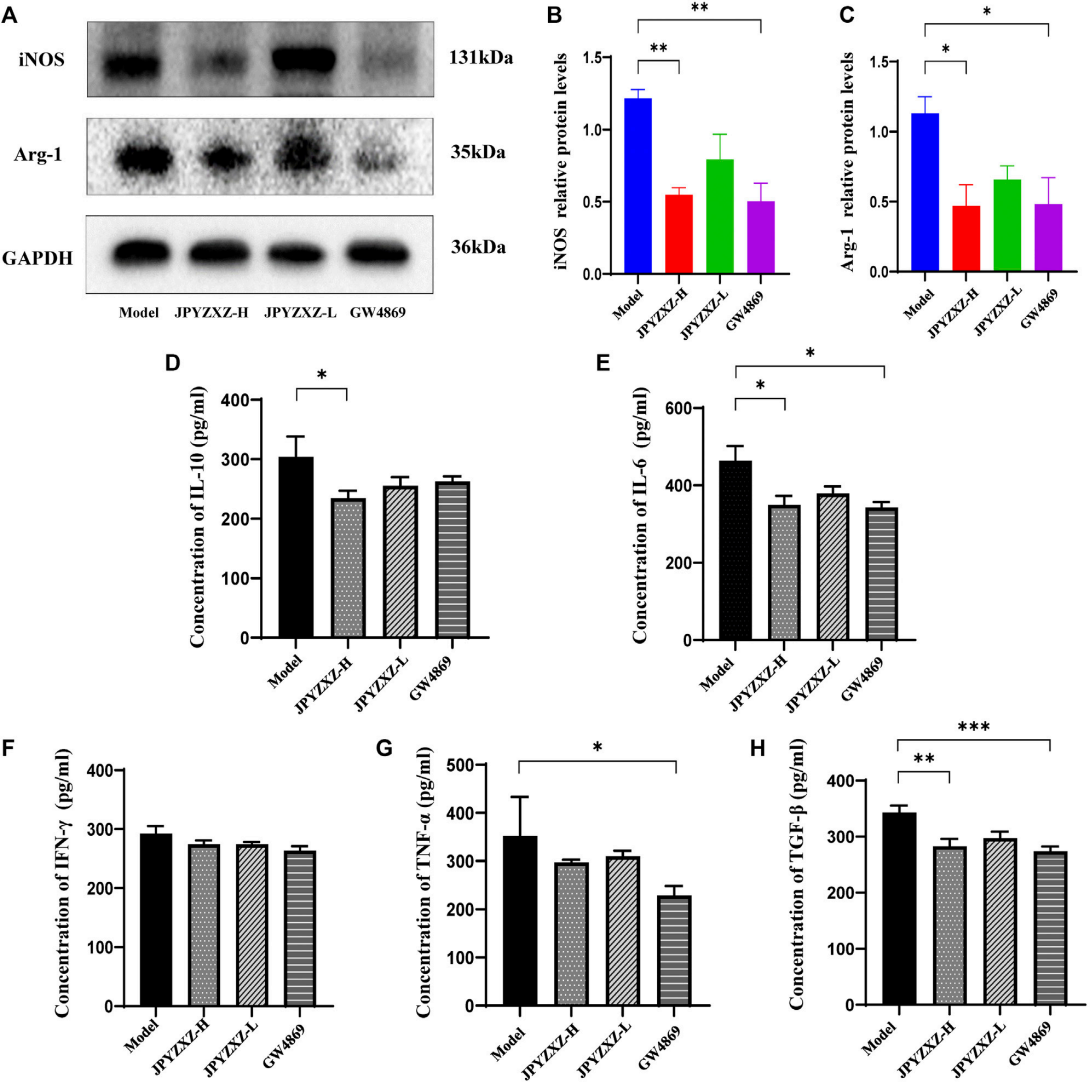
JPYZXZ reduced MDSC-related immunosuppressive cytokines in TME. **(A–C)** JPYZXZ diminished the expression levels of iNOS and Arg-1. **(D–H)** The expression levels of IL-10, IFN-γ, IL-6, TGF-β, and TNF-α in mice serum were measured using ELISA. Data are expressed as the mean ± SEM, *n* = 5 mice or *n* = 3 independent experiments. *p < 0.05, **p < 0.01, ***p < 0.001.

iNOS and Arg-1 as the main immunosuppressive factors in MDSCs were detected by Western blot, and we found that the expression of iNOS (*p* < 0.01) and Arg-1 (*p* < 0.05) were diminished in JPYZXZ-H (Figures 7A–C). Moreover, JPYZXZ significantly decreased the immunosuppression-associated cytokines IL-10 (*p* < 0.05), IL-6 (*p* < 0.05) and TGF-β (*p* < 0.01) serum levels, but not TNF-α or IFN-γ (Figures 7D–H). Altogether, it was confirmed that JPYZXZ remodeled the TME by decreasing MDSCs expansion and could reduce the expansion of MDSCs by decreasing the delivery of exosomal PD-L1 in the immunosuppressive TME.

### 3.6 JPYZXZ reduced exosomal PD-L1 in patients with GC along with the accumulation of MDSCs

**FIGURE 8:**
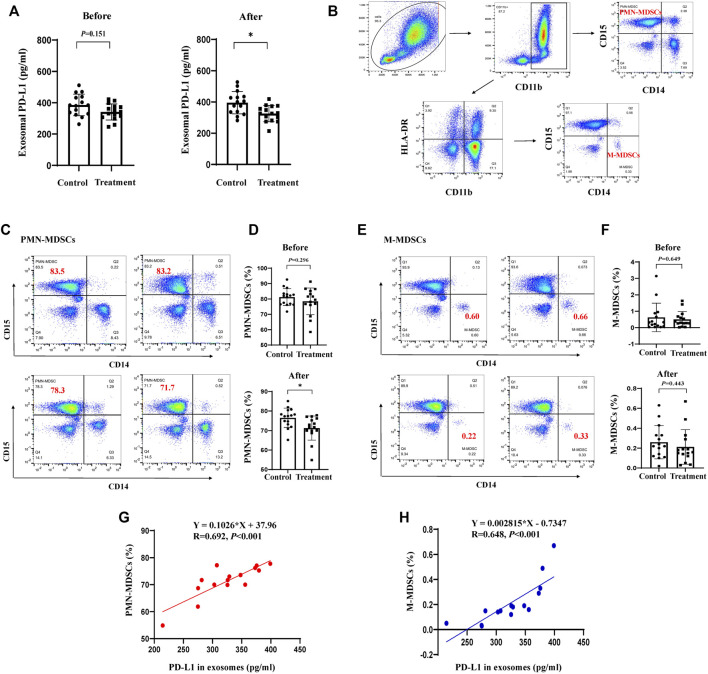
JPYZXZ reduced exosomal PD-L1, along with the proportion of MDSCs in the peripheral blood of patients with GC. **(A)** Exosomal PD-L1 content from the serum of patients with GC. **(B)** PMN-MDSCs and M-MDSCs gating process in the peripheral blood of patients with GC. Percentage of **(C,D)** PMN-MDSCs (CD11b^+^CD14^−^CD15^+^) and **(E,F)** M-MDSCs (CD11b^+^CD14^+^CD15^−^HLA-DR^-/low^) in the peripheral blood of patients with GC before and after the JPYZXZ treatment, determined using flow cytometry analysis. **(G,H)** Correlation analysis of exosomal PD-L1 and MDSCs levels after JPYZXZ treatment. Data are expressed as the mean ± SD. *p < 0.05.

To verify the clinical effect of JPYZXZ, we investigated the levels of MDSCs in peripheral blood and exosomal PD-L1 in patients with stage IIA-IIIB GC after JPYZXZ treatment. Compared with the control group, exosomal PD-L1 contents were decreased after 3 months on the JPYZXZ treatment (*p* < 0.05) (Figure 8A). Human MDSCs can be divided into polymorphonuclear (PMN-MDSCs) and monocytic (M-MDSCs). PMN-MDSCs are defined as CD11b^+^CD14^−^CD15^+^ and M-MDSCs are defined as CD11b^+^CD14^+^CD15^−^HLA-DR^-/low^ (Bronte et al., 2016) (Figure 8B). Flow cytometry analysis showed that there was a significant decrease in the levels of PMN-MDSCs in the treatment group (*p* < 0.05) (Figures 8C, D), simultaneously, there was also a decrease trend in the M-MDSCs compared to the control group (Figures 8E, F). Correlation analysis revealed a significant positive relationship between exosomal PD-L1 and PMN-MDSCs under the JPYZXZ treatment (*R* = 0.692, *p* < 0.001) (Figures 8G, H). Therefore, these data exhibited that JPYZXZ could reduce the levels of exosomal PD-L1, along with the peripheral blood PMN-MDSCs in patients with GC.

## 4 Discussion

Although immunotherapy has been increasingly used in the treatment of GC, particular attention must be paid to the low response rate for treatments targeting PD-L1 (Page et al., 2014; Fuchs et al., 2018). In the present study, we firstly found that exosomal PD-L1 of GC cells has a critical role to promote MDSCs expansion. Furthermore, it was shown that JPYZXZ could inhibit MDSCs accumulation by reducing levels of exosomal PD-L1 in GC, thereby remodeling the TME. These findings shed light on a novel mechanism of JPYZXZ in inhibiting cancer progression, and suggested the importance of exosomal PD-L1 in the treatment of GC.

As well known for hundreds of years, TCM has been widely used in China as a complementary approach to cancer treatment (Wang et al., 2018). According to the basic pathogenesis of GC, we have used the JPYZXZ decoction in GC treatment, which has shown a good efficacy in inhibiting GC progression (Wu et al., 2019) and prolonging survival in patients with advanced GC (Pan et al., 2020). Our research team previously used an herbal prescription containing the main components of JPYZXZ in treating GC, and obtained a good efficacy by reducing the expression of PD-L1 (Xu et al., 2020). In addition, recent studies indicated tumor-derived exosomes contain higher amounts of PD-L1 than that in the tumor cell membranes (Fan et al., 2019). Exosomal PD-L1 are not easily degraded by extracellular proteolytic enzymes and can participate in blood circulation more stably to reach distant organs, and are able to make contact with immunosuppressive cells in peripheral circulation at the early stage to promote distant metastasis (Chen et al., 2018; Guo et al., 2019). Furthermore, exosomal PD-L1 content in plasma samples from metastatic GC patients is correlate with their prognosis (Fan et al., 2019). Our previous study revealed that modified JPYZXZ was able to inhibit tumor progression by regulating GC-derived exosomes (Wu et al., 2022). In this study, we further detected the content of PD-L1 in the GC-derived exosomes and discussed whether it is the target of JPYZXZ. Consistently, our results showed that JPYZXZ could not only reduce PD-L1 in GC cells, but also the expression and content of PD-L1 in GC-derived exosomes. Similarly, recent research also confirmed that the removal or inhibition of exosomal PD-L1 could be used as a therapeutic adjuvant to modulate the immunosuppressive TME (Poggio et al., 2019). The above results revealed a significant implication of exosomal PD-L1 for GC treatment.

Cumulative evidence showed that tumor-derived exosomal PD-L1 is essential for communication in TME. Exosomal PD-L1 are believed to be released by tumor cells remotely impairs conventional immune surveillance by triggering the PD-1 inhibitory pathway in T cells, leading to tumor escape (Poggio et al., 2019). However, exosomal PD-L1 has been found to exert potent regulatory effect on other immunosuppressive cells. Endoplasmic reticulum (ER)-stressed oral squamous cell carcinoma cells can release PD-L1-rich exosomes, which deliver ER stress signals to infiltrating macrophages and promote malignant progression (Pang et al., 2021). Exosomal PD-L1 can disrupt the endothelial cell monolayer barrier function, increasing vascular permeability, and promoting angiogenesis, which in turn facilitates tumor cell motility and spread (Chen et al., 2018). It has also been shown that exosomal PD-L1 could be delivered to TAMs and exacerbate the immunosuppressive microenvironment in hepatocellular carcinoma (Chen et al., 2021). These studies reported here illustrated the potential regulatory functions of exosomal PD-L1 in the TME. Notably, current research has revealed a link between PD-L1 and MDSCs. MDSCs represent heterogeneous populations of immature myeloid cells that expand and play essential roles in immunosuppressive TME formation (Di Ianni et al., 2021). A recent study reported that the levels of PD-L1 expressed on the surface of MDSCs in bone marrow and spleen are significantly increased after sepsis, which aggravates the sepsis-induced immunosuppression (Ruan et al., 2020). Did exosomal PD-L1 promote MDSCs expansion in GC? In our study, we firstly found a positive correlation between PD-L1 levels and MDSCs in TISIDB, and further study revealed that GC cell-derived exosomes promoted the differentiation of MDSCs; when exosomes from GC cells overexpressing PD-L1 were added, the proportion of MDSCs were found to dramatically increase in comparison to that of exosome induction alone. After JPYZXZ treatment, the proportion of MDSCs were found to decrease in exosome-induced medium while the inhibitory effect of JPYZXZ was attenuated when PD-L1 was overexpressed. The findings demonstrated for the first time that JPYZXZ inhibited GC progression by reducing the propagation of GC-derived exosomal PD-L1, thereby suppressing the differentiation of bone marrow cells to MDSCs in mice.

The IL-6/JAK1 signaling pathway has been shown to positively regulate the stability of the PD-L1 protein and promote tumor immune escape (Chan et al., 2019). Our findings suggested that JPYZXZ reduced PD-L1 levels possibly through the inhibition of the upstream IL-6/JAK2/STAT3 signaling. Simultaneously, JPYZXZ significantly decreased the accumulation of CD11b^+^Gr-1^+^MDSCs in blood and tumor, as well as the levels of PD-L1 expressed in MDSCs. According to the results of *in vivo* experiment, we proposed that JPYZXZ inhibited tumorigenesis by reducing the level of exosomal PD-L1, thus decreasing the number of CD11b^+^Gr-1^+^MDSCs in tumors and blood. The production of iNOS and Arg-1 by MDSCs has been reported to be a major inhibitory mechanism, which mediates the formation of immunosuppressive microenvironment (Obermajer et al., 2012), we also found that JPYZXZ could decrease the expression of iNOS and Arg-1 in tumor tissues. Furthermore, inflammatory mediators accelerate the generation of immunosuppressive TME (Henrik et al., 2019). The levels of inflammatory factors TGF-β and IL-10 were downregulated by treatment with JPYZXZ. These results confirmed that exosomal PD-L1 could induce MDSCs differentiation in the TME. Meanwhile, we also confirmed that the removal or inhibition of exosomal PD-L1 can be used as a therapeutic adjuvant to modulate the immunosuppressive TME.

Of note, our clinical data showed that JPYZXZ reduced the levels of plasma exosomal PD-L1 as well as the proportion of MDSCs in peripheral blood, particularly PMN-MDSCs in patients with advanced GC, and correlation analysis revealed a positive relationship between exosomal PD-L1 and MDSCs. Similarly, studies have shown that when co-cultured with PBMCs from the human peripheral blood, PD-L1 targeting high-affinity natural killer (t-haNK) cells preferentially cleaved bone MDSCs over other immune cell types (Fabian et al., 2019). Accumulatively, these findings demonstrate for the first time that JPYZXZ inhibited tumorigenesis and progression by reducing the propagation of GC-derived exosomal PD-L1, thereby suppressing the accumulation of MDSCs. However, due to the limitation of follow-up time and sample size in clinical research, the pathological tissue of GC patients has not been analyzed, and multi-center and large sample cohort studies are still needed. In addition, some of the results did not show dose-dependent effects, potentially attributable to target receptor saturation or biological factors such as non-linear metabolism and target diversity of specific metabolites. Future research endeavors will involve expanding the dose range and conducting comprehensive investigations into the influence of the interactions among botanical drug metabolites on the overall effects of JPYZXZ decoction. Moreover, further research is needed to clarify what potential intermediate effector molecules JPYZXZ acts on exosomal PD-L1 to inhibit MDSCs expansion.

## 5 Conclusion

In summary, our study firstly demonstrated that exosomal PD-L1 could induce MDSCs expansion in GC. More importantly, we found that JPYZXZ regulated anti-tumor immunity by inhibiting the expression of PD-L1 and exosomal PD-L1, decreasing the delivery of exosomal PD-L1 to MDSCs, thus regulating the phenotype of MDSCs, remodeling the immunosuppressive TME of GC. These results help to explain the antitumor effect for JPYZXZ from an immunological perspective and identify a new mechanism by which JPYZXZ regulates MDSCs reprogramming.

## Figures and Tables

**Table:** 

**Arg-1**	Arginase-1
**BM**	Bone marrow
**CV**	Coefficients of variation
**ER**	Endoplasmic reticulum
**ESI**	Electrospray ionization
**GC**	Gastric cancer
**GM-CSF**	Granulocyte and macrophage colony-stimulating factor
**HBSS**	Hank’s buffered salt solution
**ICBs**	Immune checkpoint inhibitors
**IFN-γ**	Interferon-γ
**IL-6**	Interleukin-6
**IL-10**	Interleukin-10
**iNOS**	Inducible nitric oxide synthase
**IS**	Internal standard
**JAK2**	Janus kinase 2
**JPYZXZ**	Jianpi Yangzheng Xiaozheng decoction
**M-MDSCs**	Mononuclear myeloid-derived suppressor cells
**MDSCs**	Myeloid-derived suppressor cells
**PBMCs**	Peripheral blood mononuclear cells
**PD-L1**	Programmed cell death-ligand 1
**PMN-MDSCs**	Polymorphonuclear myeloid-derived suppressor cells
**RPA**	Relative peak area
**RRT**	Relative retention time
**STAD**	Stomach adenocarcinoma
**STAT3**	Signal transducer and activator of transcription3
**TAMs**	Tumor-associated macrophages
**TCM**	Traditional Chinese medicine
**t-haNK**	Targeting high-affinity natural killer
**TGF-β**	Transforming growth factor-β
**TME**	Tumor microenvironment

## Data Availability

The original contributions presented in the study are included in the article/Supplementary Material, further inquiries can be directed to the corresponding authors.
