# Supplementary material for: Jianpi Yangzheng Xiaozheng decoction alleviates gastric cancer progression via suppressing exosomal PD-L1
Source: Front Pharmacol. 2023 Aug 3;14:1159829. doi: 10.3389/fphar.2023.1159829 (PMC10434994; doi:10.3389/fphar.2023.1159829)
Supplement: Supplementary file 1 [file Table1.DOCX]

***Supplementary Material***

Jianpi Yangzheng Xiaozheng decoction alleviates gastric cancer progression via suppressing exosomal PD-L1

Yanzhen Chen^1,2,†^, Jiayun Liu^1,†^, Yuxuan Chen^2^, Ruijuan Zhang^2^, Jialei Tao^1^, Xu Chen^2^, Haidan Wang^1^, Qingmin Sun^1, *^, Jian Wu^1,*^, Shenlin Liu^1,*^

*^1^Jiangsu Province Hospital of Chinese Medicine, Affiliated Hospital of Nanjing University of Chinese Medicine, Nanjing, Jiangsu 210029, China*

*^2^ No. 1 Clinical Medical College, Nanjing University of Chinese Medicine, Nanjing, Jiangsu 210023, China*

† These authors have contributed equally to this work.

*Correspondence should be addressed to Shenlin Liu: liushenlin@njucm.edu.cn; Jian Wu: [jianwu@njucm.edu.cn](mailto:jianwu@njucm.edu.cn); Qingmin Sun: qingminsun@njucm.edu.cn.

**Table S1**

The herbal composition of JPYZXZ decoction.

| Herbal name | Botanical Latin name | Family | Part used | Amount used (g) |
| --- | --- | --- | --- | --- |
| Huang Qi | *Astragalus mongholicus* Bunge | Fabaceae | root | 30 |
| Dang Shen | *Codonopsis pilosula* (Franch.) Nannf. | Campanulaceae | root | 30 |
| Bai Zhu | *Atractylodes macrocephala* Koidz. | Asteraceae | rhizome | 10 |
| Dang Gui | *Angelica sinensis*（Oliv.）Diels | Apiaceae | root | 10 |
| Bai Shao | *Paeonia lactiflora* Pall. | Paeoniaceae | root | 10 |
| San Leng | *Sparganium stoloniferum* Buch.-Ham | Typhaceae | dry tuber | 30 |
| E Zhu | *Curcuma zedoria* (Christm.) Roscoe | Zingiberaceae | dry rhizome | 30 |
| Mu Xiang | *Vladimiria souliei*(Franch.) Ling | Asteraceae | root | 10 |
| Chen Pi | *Citrus reticulata* Blanco | Rutaceae | pericarp | 10 |
| Bai Hua She She Cao | *Scleromitrion diffusum*(Willd.) R.J.Wang | Rubiaceae | whole herb | 15 |
| Shi Jian Chuan | *Salvia chinensis*Benth. | Lamiaceae | whole herb | 15 |
| Gan Cao | *Glycyrrhiza uralensis* Fisch. | Fabaceae | root and rhizome | 3 |
| Total amount |  |  |  | 203 |

**Table S2**

Orthogonal experiment on preparation technology of JPYZXZ

| Number | Solid-liquid ratio (g/mL) | Soaking time/h | Boiling time/h | Decocting times |
| --- | --- | --- | --- | --- |
| S1 | 1:8 | 0.5 | 0.5 | 1 |
| S2 | 1:8 | 1 | 1 | 2 |
| S3 | 1:8 | 1.5 | 1.5 | 3 |
| S4 | 1:10 | 0.5 | 1 | 2 |
| S5 | 1:10 | 1 | 1.5 | 3 |
| S6 | 1:10 | 1.5 | 0.5 | 1 |
| S7 | 1:12 | 0.5 | 1.5 | 3 |
| S8 | 1:12 | 1 | 0.5 | 1 |
| S9 | 1:12 | 1.5 | 1 | 2 |

**Table S3**

Similarity evaluation results of ten batches of JPYZXZ samples

| **ESI^+^** |  |  |  |  |  |  |  |  |  |  |  |
| --- | --- | --- | --- | --- | --- | --- | --- | --- | --- | --- | --- |
|  | S1 | S2 | S3 | S4 | S5 | S6 | S7 | S8 | S9 | S10 | R |
| S1 | 1.000 | 0.971 | 0.971 | 0.953 | 0.972 | 0.960 | 0.961 | 0.951 | 0.932 | 0.917 | 0.979 |
| S2 | 0.971 | 1.000 | 0.973 | 0.953 | 0.978 | 0.980 | 0.974 | 0.970 | 0.951 | 0.932 | 0.988 |
| S3 | 0.971 | 0.973 | 1.000 | 0.958 | 0.966 | 0.967 | 0.952 | 0.957 | 0.954 | 0.934 | 0.982 |
| S4 | 0.953 | 0.953 | 0.958 | 1.000 | 0.943 | 0.936 | 0.930 | 0.931 | 0.931 | 0.911 | 0.964 |
| S5 | 0.972 | 0.978 | 0.966 | 0.943 | 1.000 | 0.983 | 0.988 | 0.978 | 0.963 | 0.952 | 0.991 |
| S6 | 0.960 | 0.980 | 0.967 | 0.936 | 0.983 | 1.000 | 0.984 | 0.991 | 0.974 | 0.967 | 0.992 |
| S7 | 0.961 | 0.974 | 0.952 | 0.930 | 0.988 | 0.984 | 1.000 | 0.986 | 0.966 | 0.962 | 0.988 |
| S8 | 0.951 | 0.970 | 0.957 | 0.931 | 0.978 | 0.991 | 0.986 | 1.000 | 0.977 | 0.978 | 0.989 |
| S9 | 0.932 | 0.951 | 0.954 | 0.931 | 0.963 | 0.974 | 0.966 | 0.977 | 1.000 | 0.976 | 0.978 |
| S10 | 0.917 | 0.932 | 0.934 | 0.911 | 0.952 | 0.967 | 0.962 | 0.978 | 0.976 | 1.000 | 0.967 |
| R | 0.979 | 0.988 | 0.982 | 0.964 | 0.991 | 0.992 | 0.988 | 0.989 | 0.978 | 0.967 | 1.000 |
| **ESI^−^** |  |  |  |  |  |  |  |  |  |  |  |
|  | S1 | S2 | S3 | S4 | S5 | S6 | S7 | S8 | S9 | S10 | R |
| S1 | 1.000 | 0.985 | 0.979 | 0.980 | 0.860 | 0.975 | 0.971 | 0.952 | 0.934 | 0.934 | 0.982 |
| S2 | 0.985 | 1.000 | 0.987 | 0.988 | 0.866 | 0.981 | 0.976 | 0.957 | 0.934 | 0.939 | 0.986 |
| S3 | 0.979 | 0.987 | 1.000 | 0.991 | 0.860 | 0.988 | 0.985 | 0.977 | 0.960 | 0.962 | 0.993 |
| S4 | 0.980 | 0.988 | 0.991 | 1.000 | 0.853 | 0.991 | 0.984 | 0.978 | 0.963 | 0.964 | 0.994 |
| S5 | 0.860 | 0.866 | 0.860 | 0.853 | 1.000 | 0.859 | 0.865 | 0.829 | 0.816 | 0.824 | 0.881 |
| S6 | 0.975 | 0.981 | 0.988 | 0.991 | 0.859 | 1.000 | 0.994 | 0.980 | 0.967 | 0.976 | 0.995 |
| S7 | 0.971 | 0.976 | 0.985 | 0.984 | 0.865 | 0.994 | 1.000 | 0.975 | 0.965 | 0.977 | 0.993 |
| S8 | 0.952 | 0.957 | 0.977 | 0.978 | 0.829 | 0.980 | 0.975 | 1.000 | 0.987 | 0.985 | 0.985 |
| S9 | 0.934 | 0.934 | 0.960 | 0.963 | 0.816 | 0.967 | 0.965 | 0.987 | 1.000 | 0.988 | 0.974 |
| S10 | 0.934 | 0.939 | 0.962 | 0.964 | 0.824 | 0.976 | 0.977 | 0.985 | 0.988 | 1.000 | 0.977 |
| R | 0.982 | 0.986 | 0.993 | 0.994 | 0.881 | 0.995 | 0.993 | 0.985 | 0.974 | 0.977 | 1.000 |

**Table S4**

Relative retention time of common peaks in ten batches of JPYZXZ in positive ion mode.

| **ESI^+^** | **Samples** |  |  |  |  |  |  |  |  |  |  |
| --- | --- | --- | --- | --- | --- | --- | --- | --- | --- | --- | --- |
| **Peaks** | S1 | S2 | S3 | S4 | S5 | S6 | S7 | S8 | S9 | S10 | CV% |
| P1 | 0.628 | 0.633 | 0.628 | 0.633 | 0.638 | 0.639 | 0.635 | 0.634 | 0.639 | 0.637 | 0.64% |
| P2 | 1.059 | 1.064 | 1.043 | 1.059 | 1.060 | 1.051 | 1.043 | 1.049 | 1.038 | 1.040 | 0.90% |
| P3 | 1.313 | 1.300 | 1.296 | 1.295 | 1.297 | 1.306 | 1.298 | 1.304 | 1.295 | 1.296 | 0.46% |
| P4 | 2.241 | 2.228 | 2.241 | 2.241 | 2.222 | 2.250 | 2.224 | 2.272 | 2.252 | 2.238 | 0.66% |
| P5 | 2.442 | 2.429 | 2.421 | 2.441 | 2.443 | 2.434 | 2.443 | 2.437 | 2.421 | 2.440 | 0.35% |
| P6 | 2.644 | 2.629 | 2.622 | 2.660 | 2.627 | 2.618 | 2.627 | 2.621 | 2.624 | 2.626 | 0.48% |
| P7 | 2.861 | 2.848 | 2.824 | 2.842 | 2.865 | 2.840 | 2.848 | 2.843 | 2.826 | 2.850 | 0.46% |
| P8 | 2.899 | 2.902 | 2.878 | 2.897 | 2.902 | 2.896 | 2.904 | 2.899 | 2.864 | 2.887 | 0.44% |
| P9 | 3.028 | 3.032 | 3.005 | 3.025 | 3.031 | 3.025 | 2.995 | 3.011 | 2.974 | 2.999 | 0.64% |
| P10 | 3.421 | 3.403 | 3.378 | 3.396 | 3.405 | 3.399 | 3.421 | 3.401 | 3.386 | 3.396 | 0.40% |
| P11 | 3.631 | 3.611 | 3.588 | 3.622 | 3.629 | 3.627 | 3.610 | 3.609 | 3.597 | 3.605 | 0.39% |
| P12 | 4.377 | 4.373 | 4.373 | 4.385 | 4.398 | 4.372 | 4.388 | 4.373 | 4.367 | 4.379 | 0.21% |
| P13 | 4.415 | 4.411 | 4.411 | 4.423 | 4.417 | 4.411 | 4.407 | 4.411 | 4.406 | 4.418 | 0.12% |
| P14 | 4.784 | 4.779 | 4.778 | 4.787 | 4.786 | 4.780 | 4.774 | 4.777 | 4.779 | 4.773 | 0.10% |
| P15 | 4.938 | 4.915 | 4.953 | 4.922 | 4.923 | 4.917 | 4.927 | 4.951 | 4.933 | 4.954 | 0.30% |
| P16 | 4.938 | 4.954 | 4.933 | 4.940 | 4.942 | 4.936 | 4.947 | 4.951 | 4.933 | 4.934 | 0.15% |
| P17 | 5.315 | 5.310 | 5.314 | 5.314 | 5.492 | 5.312 | 5.316 | 5.492 | 5.311 | 5.312 | 1.41% |
| P18 | 5.427 | 5.401 | 5.405 | 5.407 | 5.414 | 5.403 | 5.410 | 5.414 | 5.404 | 5.404 | 0.14% |
| P19 | 5.564 | 5.560 | 5.543 | 5.562 | 5.569 | 5.560 | 5.564 | 5.550 | 5.560 | 5.559 | 0.13% |
| P20 | 5.936 | 5.933 | 5.948 | 5.948 | 5.937 | 5.946 | 5.945 | 5.952 | 5.950 | 5.948 | 0.11% |
| P21 | 5.975 | 5.973 | 5.968 | 5.986 | 5.976 | 5.965 | 5.984 | 5.971 | 5.969 | 5.967 | 0.12% |
| P22 | 6.113 | 6.110 | 6.105 | 6.123 | 6.113 | 6.104 | 6.099 | 6.126 | 6.107 | 6.106 | 0.14% |
| P23 | 6.154 | 6.151 | 6.145 | 6.162 | 6.152 | 6.142 | 6.158 | 6.145 | 6.146 | 6.143 | 0.11% |
| P24 | 6.273 | 6.271 | 6.263 | 6.282 | 6.272 | 6.261 | 6.277 | 6.282 | 6.266 | 6.281 | 0.12% |
| P25 | 6.572 | 6.570 | 6.582 | 6.577 | 6.570 | 6.577 | 6.572 | 6.577 | 6.543 | 6.537 | 0.23% |
| P26 | 6.672 | 6.670 | 6.661 | 6.654 | 6.669 | 6.653 | 6.669 | 6.654 | 6.660 | 6.655 | 0.11% |
| P27 | 6.473 | 6.471 | 6.463 | 6.479 | 6.470 | 6.478 | 6.474 | 6.480 | 6.465 | 6.478 | 0.09% |
| P28 | 6.473 | 6.471 | 6.483 | 6.479 | 6.490 | 6.478 | 6.494 | 6.480 | 6.484 | 6.478 | 0.11% |
| P29 | 6.572 | 6.570 | 6.562 | 6.577 | 6.570 | 6.577 | 6.572 | 6.577 | 6.583 | 6.577 | 0.09% |
| P30 | 6.773 | 6.792 | 6.782 | 6.794 | 6.732 | 6.751 | 6.785 | 6.734 | 6.775 | 6.772 | 0.33% |
| P31 | 6.732 | 6.732 | 6.722 | 6.735 | 6.731 | 6.733 | 6.729 | 6.715 | 6.719 | 6.735 | 0.11% |
| P32 | 6.934 | 6.931 | 6.922 | 6.934 | 6.931 | 6.929 | 6.924 | 6.930 | 6.933 | 6.930 | 0.06% |
| P33 | 7.095 | 7.090 | 7.103 | 7.092 | 7.091 | 7.084 | 7.099 | 7.088 | 7.107 | 7.087 | 0.10% |
| P34 | 7.156 | 7.171 | 7.164 | 7.171 | 7.153 | 7.163 | 7.159 | 7.148 | 7.166 | 7.165 | 0.11% |
| P35 | 7.156 | 7.151 | 7.144 | 7.152 | 7.153 | 7.145 | 7.159 | 7.148 | 7.147 | 7.146 | 0.07% |
| P36 | 7.273 | 7.271 | 7.280 | 7.289 | 7.289 | 7.279 | 7.292 | 7.283 | 7.282 | 7.281 | 0.09% |
| P37 | 7.293 | 7.290 | 7.280 | 7.289 | 7.289 | 7.279 | 7.292 | 7.283 | 7.282 | 7.281 | 0.07% |
| P38 | 7.313 | 7.310 | 7.280 | 7.289 | 7.289 | 7.297 | 7.292 | 7.302 | 7.300 | 7.299 | 0.14% |
| P39 | 7.471 | 7.466 | 7.457 | 7.464 | 7.463 | 7.471 | 7.462 | 7.455 | 7.456 | 7.454 | 0.08% |
| P40 | 7.552 | 7.544 | 7.537 | 7.541 | 7.542 | 7.53 | 7.54 | 7.532 | 7.534 | 7.532 | 0.09% |
| P41 | 7.910 | 7.920 | 7.914 | 7.912 | 7.909 | 7.917 | 7.925 | 7.920 | 7.921 | 7.922 | 0.07% |
| P42 | 7.990 | 8.000 | 7.994 | 7.991 | 8.007 | 7.996 | 8.003 | 7.997 | 7.999 | 8.000 | 0.07% |
| P43 | 8.026 | 8.036 | 8.049 | 8.064 | 8.056 | 8.068 | 8.051 | 8.065 | 8.049 | 8.032 | 0.18% |
| P44 | 8.130 | 8.120 | 8.113 | 8.129 | 8.123 | 8.113 | 8.119 | 8.113 | 8.115 | 8.118 | 0.08% |
| P45 | 8.156 | 8.171 | 8.164 | 8.171 | 8.153 | 8.163 | 8.159 | 8.148 | 8.166 | 8.165 | 0.09% |
| P46 | 8.523 | 8.531 | 8.524 | 8.519 | 8.528 | 8.521 | 8.523 | 8.517 | 8.521 | 8.526 | 0.05% |
| P47 | 8.601 | 8.589 | 8.603 | 8.598 | 8.605 | 8.598 | 8.600 | 8.596 | 8.600 | 8.605 | 0.06% |
| P48 | 8.797 | 8.805 | 8.799 | 8.811 | 8.816 | 8.811 | 8.813 | 8.807 | 8.813 | 8.799 | 0.08% |
| P49 | 9.011 | 9.020 | 9.014 | 9.026 | 9.028 | 9.022 | 9.026 | 9.019 | 9.028 | 9.012 | 0.07% |
| P50 | 9.070 | 9.057 | 9.052 | 9.064 | 9.067 | 9.061 | 9.064 | 9.058 | 9.065 | 9.052 | 0.07% |
| P51 | 9.120 | 9.102 | 9.118 | 9.128 | 9.110 | 9.124 | 9.127 | 9.117 | 9.126 | 9.117 | 0.09% |
| P52 | 9.170 | 9.154 | 9.150 | 9.162 | 9.164 | 9.157 | 9.160 | 9.154 | 9.162 | 9.150 | 0.07% |
| P53 | 9.228 | 9.213 | 9.209 | 9.240 | 9.222 | 9.215 | 9.218 | 9.211 | 9.200 | 9.207 | 0.12% |
| P54 | 9.372 | 9.360 | 9.375 | 9.367 | 9.370 | 9.365 | 9.368 | 9.360 | 9.369 | 9.374 | 0.06% |
| P55 | 9.443 | 9.446 | 9.441 | 9.451 | 9.453 | 9.447 | 9.449 | 9.442 | 9.450 | 9.441 | 0.05% |
| P56 | 9.531 | 9.493 | 9.527 | 9.515 | 9.515 | 9.492 | 9.494 | 9.503 | 9.494 | 9.507 | 0.15% |
| P57 | 9.501 | 9.499 | 9.510 | 9.512 | 9.494 | 9.491 | 9.492 | 9.500 | 9.491 | 9.507 | 0.08% |
| P58 | 9.637 | 9.639 | 9.653 | 9.642 | 9.625 | 9.620 | 9.640 | 9.632 | 9.623 | 9.633 | 0.10% |
| P59 | 9.579 | 9.581 | 9.576 | 9.566 | 9.586 | 9.581 | 9.583 | 9.576 | 9.565 | 9.575 | 0.07% |
| P60 | 9.772 | 9.754 | 9.769 | 9.777 | 9.758 | 9.753 | 9.756 | 9.766 | 9.755 | 9.766 | 0.09% |
| P61 | 9.754 | 9.754 | 9.750 | 9.758 | 9.758 | 9.753 | 9.756 | 9.746 | 9.755 | 9.746 | 0.04% |
| P62 | 9.754 | 9.754 | 9.750 | 9.758 | 9.739 | 9.753 | 9.737 | 9.746 | 9.755 | 9.746 | 0.07% |
| P63 | 9.879 | 9.896 | 9.887 | 9.890 | 9.891 | 9.886 | 9.887 | 9.895 | 9.886 | 9.904 | 0.07% |
| P64 | 9.860 | 9.882 | 9.884 | 9.870 | 9.865 | 9.875 | 9.873 | 9.841 | 9.875 | 9.853 | 0.14% |
| P65 | 9.871 | 9.873 | 9.865 | 9.874 | 9.874 | 9.870 | 9.872 | 9.862 | 9.871 | 9.865 | 0.04% |
| P66 | 10.339 | 10.337 | 10.329 | 10.331 | 10.333 | 10.329 | 10.331 | 10.338 | 10.329 | 10.327 | 0.04% |
| P67 | 10.495 | 10.492 | 10.483 | 10.485 | 10.487 | 10.500 | 10.502 | 10.491 | 10.500 | 10.500 | 0.07% |
| P68 | 10.519 | 10.514 | 10.520 | 10.521 | 10.527 | 10.511 | 10.513 | 10.501 | 10.513 | 10.518 | 0.07% |
| P69 | 10.026 | 10.008 | 10.021 | 10.027 | 10.026 | 10.004 | 10.006 | 10.014 | 10.006 | 10.018 | 0.09% |
| P70 | 10.695 | 10.692 | 10.683 | 10.685 | 10.687 | 10.681 | 10.683 | 10.653 | 10.681 | 10.662 | 0.12% |
| P71 | 10.671 | 10.668 | 10.676 | 10.658 | 10.662 | 10.672 | 10.673 | 10.662 | 10.673 | 10.673 | 0.06% |
| P72 | 10.806 | 10.803 | 10.813 | 10.811 | 10.817 | 10.805 | 10.808 | 10.812 | 10.805 | 10.806 | 0.04% |
| P73 | 11.004 | 11.000 | 11.007 | 11.006 | 10.995 | 11.000 | 11.003 | 11.008 | 11.000 | 11.003 | 0.04% |
| P74 | 11.102 | 11.097 | 11.104 | 11.103 | 11.111 | 11.095 | 11.098 | 11.105 | 11.116 | 11.102 | 0.06% |
| P75 | 11.180 | 11.175 | 11.181 | 11.182 | 11.189 | 11.172 | 11.175 | 11.182 | 11.174 | 11.179 | 0.05% |
| P76 | 11.219 | 11.214 | 11.220 | 11.221 | 11.227 | 11.230 | 11.232 | 11.221 | 11.232 | 11.218 | 0.06% |
| P77 | 11.258 | 11.252 | 11.259 | 11.258 | 11.247 | 11.249 | 11.250 | 11.259 | 11.251 | 11.257 | 0.04% |
| P78 | 11.719 | 11.733 | 11.720 | 11.740 | 11.727 | 11.749 | 11.732 | 11.721 | 11.732 | 11.737 | 0.08% |
| P79 | 11.832 | 11.826 | 11.829 | 11.831 | 11.818 | 11.818 | 11.822 | 11.828 | 11.819 | 11.828 | 0.05% |
| P80 | 11.959 | 11.950 | 11.955 | 11.955 | 11.963 | 11.958 | 11.961 | 11.951 | 11.963 | 11.957 | 0.04% |
| P81 | 12.018 | 12.009 | 12.014 | 12.013 | 12.020 | 12.016 | 12.018 | 12.008 | 12.022 | 12.016 | 0.04% |
| P82 | 12.004 | 11.797 | 12.000 | 12.002 | 12.007 | 12.006 | 12.008 | 11.797 | 12.009 | 12.001 | 0.73% |
| P83 | 12.094 | 12.085 | 12.090 | 12.089 | 12.095 | 12.092 | 12.092 | 12.085 | 12.078 | 12.091 | 0.04% |
| P84 | 12.502 | 12.492 | 12.497 | 12.493 | 12.501 | 12.493 | 12.494 | 12.488 | 12.498 | 12.497 | 0.03% |
| P85 | 12.511 | 12.521 | 12.505 | 12.524 | 12.511 | 12.507 | 12.526 | 12.518 | 12.512 | 12.506 | 0.06% |
| P86 | 12.598 | 12.609 | 12.612 | 12.61 | 12.598 | 12.610 | 12.610 | 12.604 | 12.614 | 12.616 | 0.05% |
| P87 | 12.677 | 12.687 | 12.671 | 12.687 | 12.696 | 12.686 | 12.688 | 12.682 | 12.691 | 12.675 | 0.06% |
| P88 | 13.404 | 12.695 | 12.695 | 12.688 | 12.600 | 12.684 | 12.687 | 12.683 | 12.694 | 12.688 | 1.81% |
| P89 | 12.737 | 12.746 | 12.750 | 12.747 | 12.755 | 12.744 | 12.746 | 12.740 | 12.750 | 12.755 | 0.05% |
| P90 | 13.148 | 13.157 | 13.139 | 13.174 | 13.143 | 13.150 | 13.153 | 13.149 | 13.159 | 13.149 | 0.07% |
| P91 | 13.404 | 13.395 | 13.395 | 13.388 | 13.400 | 13.384 | 13.387 | 13.383 | 13.394 | 13.388 | 0.05% |
| P92 | 13.423 | 13.414 | 13.414 | 13.428 | 13.439 | 13.423 | 13.427 | 13.422 | 13.414 | 13.428 | 0.06% |
| P93 | 13.500 | 13.489 | 13.491 | 13.505 | 13.496 | 13.498 | 13.503 | 13.498 | 13.491 | 13.486 | 0.05% |
| P94 | 14.516 | 14.506 | 14.514 | 14.518 | 14.507 | 14.501 | 14.516 | 14.508 | 14.519 | 14.515 | 0.04% |
| P95 | 14.789 | 14.778 | 14.783 | 14.770 | 14.777 | 14.772 | 14.789 | 14.758 | 14.791 | 14.773 | 0.07% |

**Table S5**

Relative retention time of common peaks in ten batches of JPYZXZ in negative ion mode.

| **ESI^−^** | **Samples** |  |  |  |  |  |  |  |  |  |  |
| --- | --- | --- | --- | --- | --- | --- | --- | --- | --- | --- | --- |
| **Peaks** | S1 | S2 | S3 | S4 | S5 | S6 | S7 | S8 | S9 | S10 | CV% |
| P1 | 1.077 | 1.086 | 1.085 | 1.088 | 1.066 | 1.077 | 1.077 | 1.088 | 1.077 | 1.077 | 0.64% |
| P2 | 1.138 | 1.145 | 1.134 | 1.154 | 1.179 | 1.140 | 1.192 | 1.172 | 1.143 | 1.159 | 1.69% |
| P3 | 1.350 | 1.359 | 1.358 | 1.351 | 1.348 | 1.344 | 1.342 | 1.354 | 1.354 | 1.343 | 0.45% |
| P4 | 1.405 | 1.413 | 1.413 | 1.406 | 1.359 | 1.399 | 1.408 | 1.409 | 1.409 | 1.398 | 1.13% |
| P5 | 1.438 | 1.445 | 1.445 | 1.449 | 1.437 | 1.431 | 1.429 | 1.452 | 1.441 | 1.430 | 0.56% |
| P6 | 1.911 | 1.908 | 1.906 | 1.901 | 1.956 | 1.906 | 1.904 | 1.906 | 1.898 | 1.898 | 0.89% |
| P7 | 2.076 | 2.072 | 2.082 | 2.066 | 2.113 | 2.062 | 2.059 | 2.086 | 2.075 | 2.055 | 0.81% |
| P8 | 2.399 | 2.385 | 2.405 | 2.391 | 2.401 | 2.398 | 2.395 | 2.411 | 2.401 | 2.393 | 0.31% |
| P9 | 2.768 | 2.763 | 2.764 | 2.762 | 2.805 | 2.757 | 2.755 | 2.772 | 2.760 | 2.755 | 0.53% |
| P10 | 3.806 | 3.806 | 3.806 | 3.807 | 3.819 | 3.815 | 3.814 | 3.815 | 3.809 | 3.809 | 0.12% |
| P11 | 3.895 | 3.894 | 3.884 | 3.886 | 3.887 | 3.892 | 3.883 | 3.883 | 3.876 | 3.878 | 0.16% |
| P12 | 4.016 | 4.015 | 4.017 | 4.008 | 4.001 | 4.004 | 4.004 | 4.005 | 3.999 | 3.989 | 0.22% |
| P13 | 4.138 | 4.126 | 4.127 | 4.140 | 4.147 | 4.114 | 4.139 | 4.116 | 4.133 | 4.124 | 0.26% |
| P14 | 4.680 | 4.678 | 4.670 | 4.672 | 4.675 | 4.673 | 4.676 | 4.675 | 4.670 | 4.663 | 0.10% |
| P15 | 4.712 | 4.711 | 4.703 | 4.704 | 4.710 | 4.707 | 4.709 | 4.708 | 4.703 | 4.709 | 0.07% |
| P16 | 5.418 | 5.417 | 5.406 | 5.411 | 5.430 | 5.409 | 5.409 | 5.407 | 5.404 | 5.401 | 0.16% |
| P17 | 5.582 | 5.580 | 5.571 | 5.575 | 5.577 | 5.586 | 5.574 | 5.572 | 5.569 | 5.567 | 0.11% |
| P18 | 5.723 | 5.722 | 5.724 | 5.685 | 5.757 | 5.707 | 5.718 | 5.725 | 5.712 | 5.712 | 0.32% |
| P19 | 5.888 | 5.886 | 5.877 | 5.881 | 5.903 | 5.872 | 5.881 | 5.868 | 5.866 | 5.866 | 0.20% |
| P20 | 5.910 | 5.907 | 5.909 | 5.914 | 5.914 | 5.904 | 5.903 | 5.911 | 5.909 | 5.909 | 0.06% |
| P21 | 6.019 | 6.016 | 6.009 | 6.023 | 6.071 | 6.025 | 6.025 | 6.011 | 6.009 | 6.020 | 0.30% |
| P22 | 6.152 | 6.148 | 6.143 | 6.145 | 6.139 | 6.137 | 6.136 | 6.145 | 6.134 | 6.132 | 0.11% |
| P23 | 6.263 | 6.259 | 6.254 | 6.256 | 6.276 | 6.261 | 6.269 | 6.267 | 6.256 | 6.254 | 0.12% |
| P24 | 6.384 | 6.392 | 6.376 | 6.378 | 6.424 | 6.384 | 6.381 | 6.378 | 6.367 | 6.377 | 0.24% |
| P25 | 6.394 | 6.392 | 6.387 | 6.389 | 6.390 | 6.384 | 6.381 | 6.378 | 6.367 | 6.377 | 0.13% |
| P26 | 6.448 | 6.446 | 6.453 | 6.443 | 6.448 | 6.449 | 6.447 | 6.443 | 6.433 | 6.431 | 0.11% |
| P27 | 6.581 | 6.578 | 6.574 | 6.576 | 6.606 | 6.570 | 6.568 | 6.565 | 6.554 | 6.565 | 0.21% |
| P28 | 6.614 | 6.611 | 6.608 | 6.609 | 6.606 | 6.604 | 6.603 | 6.598 | 6.598 | 6.599 | 0.08% |
| P29 | 7.227 | 7.237 | 7.231 | 7.234 | 7.245 | 7.234 | 7.232 | 7.236 | 7.229 | 7.240 | 0.07% |
| P30 | 7.370 | 7.367 | 7.362 | 7.365 | 7.369 | 7.366 | 7.364 | 7.368 | 7.360 | 7.362 | 0.04% |
| P31 | 7.643 | 7.641 | 7.637 | 7.629 | 7.683 | 7.642 | 7.630 | 7.623 | 7.614 | 7.619 | 0.25% |
| P32 | 7.741 | 7.738 | 7.734 | 7.738 | 7.739 | 7.739 | 7.739 | 7.732 | 7.723 | 7.728 | 0.08% |
| P33 | 8.013 | 8.011 | 8.008 | 8.002 | 8.020 | 8.004 | 8.001 | 7.996 | 7.988 | 7.995 | 0.12% |
| P34 | 8.056 | 8.054 | 8.051 | 8.056 | 8.054 | 8.047 | 8.044 | 8.050 | 8.043 | 8.038 | 0.08% |
| P35 | 8.067 | 8.065 | 8.072 | 8.067 | 8.064 | 8.069 | 8.067 | 8.071 | 8.065 | 8.060 | 0.04% |
| P36 | 8.165 | 8.163 | 8.159 | 8.154 | 8.155 | 8.157 | 8.166 | 8.148 | 8.142 | 8.147 | 0.10% |
| P37 | 8.454 | 8.450 | 8.447 | 8.452 | 8.503 | 8.445 | 8.453 | 8.426 | 8.440 | 8.435 | 0.24% |
| P38 | 8.408 | 8.405 | 8.401 | 8.408 | 8.405 | 8.412 | 8.410 | 8.404 | 8.408 | 8.403 | 0.04% |
| P39 | 8.751 | 8.747 | 8.744 | 8.741 | 8.786 | 8.744 | 8.743 | 8.792 | 8.784 | 8.737 | 0.24% |
| P40 | 8.804 | 8.812 | 8.798 | 8.806 | 8.812 | 8.798 | 8.797 | 8.792 | 8.796 | 8.794 | 0.08% |
| P41 | 8.951 | 8.946 | 8.946 | 8.941 | 8.962 | 8.944 | 8.934 | 8.939 | 8.931 | 8.930 | 0.11% |
| P42 | 9.147 | 9.155 | 9.155 | 9.161 | 9.178 | 9.154 | 9.155 | 9.127 | 9.129 | 9.141 | 0.17% |
| P43 | 9.279 | 9.276 | 9.276 | 9.271 | 9.278 | 9.274 | 9.265 | 9.270 | 9.262 | 9.263 | 0.07% |
| P44 | 9.470 | 9.466 | 9.455 | 9.483 | 9.508 | 9.477 | 9.482 | 9.462 | 9.487 | 9.453 | 0.18% |
| P45 | 9.526 | 9.534 | 9.523 | 9.529 | 9.566 | 9.523 | 9.528 | 9.520 | 9.509 | 9.510 | 0.17% |
| P46 | 9.836 | 9.833 | 9.833 | 9.840 | 9.838 | 9.836 | 9.838 | 9.832 | 9.833 | 9.835 | 0.03% |
| P47 | 10.267 | 10.274 | 10.264 | 10.249 | 10.291 | 10.256 | 10.260 | 10.288 | 10.245 | 10.248 | 0.16% |
| P48 | 10.344 | 10.341 | 10.329 | 10.327 | 10.361 | 10.336 | 10.328 | 10.322 | 10.323 | 10.316 | 0.13% |
| P49 | 10.356 | 10.352 | 10.351 | 10.350 | 10.383 | 10.347 | 10.339 | 10.334 | 10.335 | 10.339 | 0.14% |
| P50 | 10.488 | 10.484 | 10.484 | 10.482 | 10.518 | 10.480 | 10.472 | 10.466 | 10.455 | 10.461 | 0.17% |
| P51 | 10.488 | 10.484 | 10.484 | 10.494 | 10.484 | 10.490 | 10.495 | 10.489 | 10.489 | 10.484 | 0.04% |
| P52 | 10.877 | 10.876 | 10.875 | 10.884 | 10.896 | 10.883 | 10.877 | 10.869 | 10.871 | 10.878 | 0.07% |
| P53 | 10.966 | 10.964 | 10.964 | 10.962 | 10.975 | 10.973 | 10.967 | 10.969 | 10.961 | 10.968 | 0.04% |
| P54 | 11.010 | 11.009 | 11.009 | 11.007 | 11.010 | 11.007 | 11.013 | 11.003 | 11.007 | 11.003 | 0.03% |
| P55 | 11.055 | 11.055 | 11.054 | 11.051 | 11.078 | 11.052 | 11.047 | 11.037 | 11.030 | 11.036 | 0.12% |
| P56 | 11.144 | 11.145 | 11.143 | 11.142 | 11.158 | 11.132 | 11.137 | 11.127 | 11.121 | 11.116 | 0.11% |
| P57 | 11.190 | 11.191 | 11.177 | 11.177 | 11.193 | 11.177 | 11.182 | 11.172 | 11.166 | 11.161 | 0.10% |
| P58 | 11.247 | 11.248 | 11.247 | 11.245 | 11.252 | 11.245 | 11.250 | 11.241 | 11.246 | 11.240 | 0.03% |
| P59 | 11.480 | 11.482 | 11.479 | 11.480 | 11.480 | 11.470 | 11.474 | 11.464 | 11.459 | 11.464 | 0.07% |
| P60 | 11.525 | 11.528 | 11.524 | 11.525 | 11.525 | 11.527 | 11.530 | 11.520 | 11.526 | 11.521 | 0.03% |
| P61 | 12.161 | 12.155 | 12.159 | 12.160 | 12.165 | 12.165 | 12.155 | 12.159 | 12.154 | 12.161 | 0.03% |
| P62 | 12.331 | 12.337 | 12.331 | 12.341 | 12.345 | 12.337 | 12.339 | 12.343 | 12.338 | 12.335 | 0.04% |
| P63 | 12.377 | 12.370 | 12.375 | 12.374 | 12.368 | 12.371 | 12.372 | 12.366 | 12.373 | 12.369 | 0.03% |
| P64 | 12.388 | 12.382 | 12.375 | 12.374 | 12.379 | 12.382 | 12.372 | 12.377 | 12.373 | 12.369 | 0.05% |
| P65 | 12.501 | 12.494 | 12.488 | 12.499 | 12.496 | 12.496 | 12.485 | 12.491 | 12.476 | 12.472 | 0.08% |
| P66 | 12.512 | 12.506 | 12.500 | 12.499 | 12.543 | 12.496 | 12.496 | 12.491 | 12.487 | 12.484 | 0.13% |
| P67 | 12.580 | 12.574 | 12.578 | 12.577 | 12.577 | 12.564 | 12.565 | 12.559 | 12.556 | 12.553 | 0.08% |
| P68 | 12.840 | 12.845 | 12.829 | 12.838 | 12.835 | 12.825 | 12.826 | 12.820 | 12.816 | 12.814 | 0.08% |
| P69 | 13.684 | 13.693 | 13.683 | 13.688 | 13.688 | 13.676 | 13.675 | 13.680 | 13.674 | 13.673 | 0.05% |
| P70 | 13.793 | 13.790 | 13.780 | 13.783 | 13.784 | 13.784 | 13.783 | 13.778 | 13.782 | 13.781 | 0.03% |
| P71 | 13.879 | 13.876 | 13.878 | 13.876 | 13.876 | 13.871 | 13.882 | 13.874 | 13.884 | 13.870 | 0.03% |
| P72 | 13.902 | 13.899 | 13.901 | 13.888 | 13.900 | 13.894 | 13.882 | 13.886 | 13.884 | 13.881 | 0.06% |
| P73 | 13.925 | 13.934 | 13.924 | 13.923 | 13.924 | 13.918 | 13.917 | 13.921 | 13.907 | 13.904 | 0.06% |
| P74 | 14.053 | 14.050 | 14.052 | 14.053 | 14.054 | 14.046 | 14.057 | 14.049 | 14.048 | 14.044 | 0.03% |
| P75 | 14.124 | 14.131 | 14.122 | 14.125 | 14.113 | 14.117 | 14.093 | 14.108 | 14.096 | 14.091 | 0.10% |
| P76 | 14.228 | 14.224 | 14.227 | 14.229 | 14.231 | 14.221 | 14.220 | 14.222 | 14.225 | 14.218 | 0.03% |
| P77 | 14.415 | 14.423 | 14.414 | 14.418 | 14.420 | 14.420 | 14.418 | 14.421 | 14.412 | 14.416 | 0.02% |
| P78 | 15.100 | 15.104 | 15.094 | 15.098 | 15.103 | 15.101 | 15.085 | 15.100 | 15.093 | 15.094 | 0.04% |
| P79 | 15.171 | 15.173 | 15.175 | 15.169 | 15.173 | 15.172 | 15.165 | 15.157 | 15.174 | 15.175 | 0.04% |
| P80 | 15.229 | 15.231 | 15.232 | 15.239 | 15.234 | 15.229 | 15.246 | 15.239 | 15.231 | 15.221 | 0.05% |
| P81 | 15.370 | 15.360 | 15.349 | 15.356 | 15.388 | 15.349 | 15.354 | 15.334 | 15.337 | 15.339 | 0.11% |
| P82 | 15.510 | 15.502 | 15.502 | 15.509 | 15.507 | 15.501 | 15.507 | 15.509 | 15.501 | 15.504 | 0.02% |
| P83 | 15.531 | 15.535 | 15.535 | 15.531 | 15.528 | 15.534 | 15.528 | 15.543 | 15.534 | 15.525 | 0.03% |
| P84 | 16.113 | 16.115 | 16.112 | 16.120 | 16.109 | 16.115 | 16.121 | 16.120 | 16.112 | 16.114 | 0.02% |
| P85 | 16.337 | 16.338 | 16.333 | 16.332 | 16.333 | 16.338 | 16.333 | 16.341 | 16.333 | 16.336 | 0.02% |
| P86 | 16.594 | 16.595 | 16.588 | 16.599 | 16.603 | 16.595 | 16.600 | 16.598 | 16.590 | 16.593 | 0.03% |
| P87 | 17.115 | 17.114 | 17.118 | 17.121 | 17.112 | 17.118 | 17.119 | 17.116 | 17.111 | 17.113 | 0.02% |
| P88 | 17.345 | 17.345 | 17.346 | 17.351 | 17.344 | 17.349 | 17.349 | 17.346 | 17.353 | 17.354 | 0.02% |
| P89 | 17.880 | 17.866 | 17.867 | 17.874 | 17.859 | 17.862 | 17.860 | 17.858 | 17.857 | 17.854 | 0.05% |

**Table S6**

Relative peak areas of common peaks in ten batches of JPYZXZ in positive ion mode.

| **ESI^+^** | **Samples** |  |  |  |  |  |  |  |  |  |  |
| --- | --- | --- | --- | --- | --- | --- | --- | --- | --- | --- | --- |
| **Peaks** | S1 | S2 | S3 | S4 | S5 | S6 | S7 | S8 | S9 | S10 | CV% |
| P1 | 0.2627 | 0.2790 | 0.2183 | 0.0651 | 0.2876 | 0.3005 | 0.2790 | 0.2540 | 0.1877 | 0.1619 | 31.97% |
| P2 | 0.0307 | 2.2341 | 1.9715 | 0.7417 | 0.0421 | 0.0506 | 0.0555 | 0.0477 | 1.4275 | 2.6542 | 113.63% |
| P3 | 0.6175 | 0.3365 | 0.3080 | 0.5501 | 0.2887 | 0.6911 | 0.5652 | 0.4435 | 0.3590 | 0.6679 | 31.91% |
| P4 | 3.8892 | 4.1300 | 2.5356 | 5.6958 | 6.2316 | 5.0735 | 5.8287 | 4.2415 | 2.9812 | 2.3094 | 32.61% |
| P5 | 11.2862 | 12.4116 | 10.5103 | 12.7332 | 0.0178 | 13.1940 | 11.3115 | 0.0137 | 8.9619 | 8.6763 | 55.16% |
| P6 | 0.1281 | 0.1214 | 0.1225 | 1.1230 | 0.1255 | 0.1178 | 0.0579 | 0.2392 | 0.0966 | 0.0458 | 147.97% |
| P7 | 0.2623 | 0.3931 | 0.2602 | 0.3869 | 0.2547 | 0.2509 | 0.2080 | 0.1884 | 0.1883 | 0.0685 | 38.70% |
| P8 | 1.7941 | 1.9486 | 1.4414 | 1.9558 | 1.6980 | 1.6204 | 1.4749 | 1.3818 | 0.9089 | 0.6269 | 29.06% |
| P9 | 0.3609 | 0.4411 | 0.0984 | 0.2491 | 0.4977 | 0.5745 | 0.6863 | 0.5272 | 0.4988 | 0.0666 | 51.09% |
| P10 | 0.4759 | 0.5488 | 0.5069 | 0.5756 | 0.5318 | 0.5308 | 0.5438 | 0.5311 | 0.5356 | 0.4474 | 7.11% |
| P11 | 0.1884 | 0.1997 | 0.1797 | 0.2403 | 0.1690 | 0.1391 | 0.1219 | 0.1097 | 0.0690 | 0.0358 | 43.08% |
| P12 | 0.0718 | 0.0642 | 0.0746 | 0.0754 | 0.0662 | 0.0463 | 0.0468 | 0.0494 | 0.0325 | 0.0286 | 30.98% |
| P13 | 0.1130 | 0.1042 | 0.1068 | 0.1372 | 0.1170 | 0.0772 | 0.0769 | 0.0793 | 0.0777 | 0.0596 | 25.47% |
| P14 | 0.0467 | 0.0582 | 0.0476 | 0.0637 | 0.0398 | 0.0290 | 0.0269 | 0.0289 | 0.0233 | 0.0119 | 43.67% |
| P15 | 0.0309 | 0.0356 | 0.0248 | 0.0346 | 0.0330 | 0.0229 | 0.0183 | 0.0252 | 0.0170 | 0.0132 | 30.69% |
| P16 | 0.0911 | 0.0920 | 0.0801 | 0.1102 | 0.1024 | 0.1042 | 0.1039 | 0.1168 | 0.1158 | 0.1112 | 11.53% |
| P17 | 0.0668 | 0.0844 | 0.0706 | 0.0929 | 0.0872 | 0.0803 | 0.0716 | 0.0852 | 0.0680 | 0.0783 | 11.43% |
| P18 | 0.1143 | 0.1229 | 0.1011 | 0.1351 | 0.0939 | 0.0774 | 0.0636 | 0.0600 | 0.0513 | 0.0300 | 40.09% |
| P19 | 0.0558 | 0.0485 | 0.0594 | 0.0150 | 0.0564 | 0.0475 | 0.0400 | 0.0374 | 0.0366 | 0.0274 | 33.08% |
| P20 | 0.0705 | 0.0754 | 0.0937 | 0.0905 | 0.0818 | 0.0795 | 0.0553 | 0.0595 | 0.0565 | 0.0531 | 20.88% |
| P21 | 0.0313 | 0.0247 | 0.0268 | 0.0314 | 0.0202 | 0.0173 | 0.0155 | 0.0143 | 0.0157 | 0.0066 | 39.45% |
| P22 | 0.0890 | 0.0859 | 0.0743 | 0.0876 | 0.0587 | 0.0573 | 0.0485 | 0.0446 | 0.0345 | 0.0185 | 40.24% |
| P23 | 0.0404 | 0.0389 | 0.0373 | 0.0438 | 0.0297 | 0.0299 | 0.0280 | 0.0238 | 0.0211 | 0.0122 | 32.17% |
| P24 | 0.4966 | 0.5141 | 0.3994 | 0.4637 | 0.3659 | 0.3471 | 0.2732 | 0.2438 | 0.2156 | 0.1257 | 37.39% |
| P25 | 1.2732 | 1.1736 | 1.1110 | 1.5569 | 1.1397 | 1.3048 | 1.3023 | 1.3888 | 1.0334 | 0.6243 | 20.96% |
| P26 | 0.1718 | 0.1690 | 0.1484 | 0.2194 | 0.2020 | 0.2290 | 0.2258 | 0.2017 | 0.1910 | 0.1805 | 13.74% |
| P27 | 0.0338 | 0.0265 | 0.0242 | 0.0300 | 0.0207 | 0.0194 | 0.0165 | 0.0139 | 0.0148 | 0.0089 | 37.16% |
| P28 | 0.0416 | 0.0393 | 0.0376 | 0.0486 | 0.0336 | 0.0368 | 0.0309 | 0.0251 | 0.0251 | 0.0166 | 27.92% |
| P29 | 0.0494 | 0.0514 | 0.0442 | 0.0584 | 0.0411 | 0.0385 | 0.0387 | 0.0269 | 0.0291 | 0.0217 | 29.01% |
| P30 | 0.0844 | 0.0912 | 0.0022 | 0.0049 | 0.0071 | 0.0086 | 0.0101 | 0.0102 | 0.1062 | 0.0690 | 108.08% |
| P31 | 1.1073 | 1.1527 | 0.9107 | 1.3166 | 0.8900 | 0.7914 | 0.7152 | 0.6200 | 0.5552 | 0.3402 | 35.52% |
| P32 | 0.3211 | 0.3322 | 0.2180 | 0.3996 | 0.3177 | 0.4143 | 0.3839 | 0.3473 | 0.1171 | 0.2276 | 30.41% |
| P33 | 0.2755 | 0.3135 | 0.2358 | 0.3565 | 0.2631 | 0.1570 | 0.1792 | 0.1708 | 0.1085 | 0.1089 | 39.27% |
| P34 | 0.2942 | 0.3008 | 0.3289 | 0.4401 | 0.3521 | 0.4343 | 0.3750 | 0.2352 | 0.3669 | 0.2143 | 22.57% |
| P35 | 0.1856 | 0.2197 | 0.1160 | 0.1917 | 0.1928 | 0.1648 | 0.0124 | 0.1627 | 0.0747 | 0.0723 | 48.31% |
| P36 | 1.2913 | 1.3621 | 1.3690 | 1.3473 | 1.3754 | 1.3837 | 1.3116 | 1.2727 | 1.3399 | 1.2907 | 2.99% |
| P37 | 0.1967 | 0.2463 | 0.2220 | 0.2623 | 0.2345 | 0.2475 | 0.2532 | 0.2640 | 0.3848 | 0.2969 | 19.57% |
| P38 | 1.8909 | 2.0800 | 1.5255 | 2.2167 | 1.8188 | 2.8366 | 3.6239 | 2.4386 | 1.9689 | 2.2845 | 26.34% |
| P39 | 0.5535 | 0.6649 | 0.5142 | 0.8370 | 0.8472 | 0.6195 | 0.6075 | 0.5707 | 0.6089 | 0.4496 | 20.41% |
| P40 | 0.1069 | 0.1153 | 0.0958 | 0.1564 | 0.5908 | 0.1148 | 0.1355 | 0.1133 | 0.1292 | 0.0847 | 92.05% |
| P41 | 0.1305 | 0.1303 | 0.0088 | 0.1513 | 0.0763 | 0.0921 | 0.0794 | 0.0705 | 0.0552 | 0.0392 | 53.12% |
| P42 | 0.5137 | 0.3637 | 0.4737 | 0.6848 | 0.3358 | 0.4142 | 0.3884 | 0.3821 | 0.4156 | 0.3185 | 25.05% |
| P43 | 0.0387 | 0.0326 | 0.0343 | 0.0610 | 0.0404 | 0.1108 | 0.0657 | 0.0442 | 0.0435 | 0.0355 | 47.03% |
| P44 | 0.0085 | 0.0105 | 0.0348 | 0.0112 | 0.0793 | 0.0591 | 0.0576 | 0.0550 | 0.0452 | 0.0066 | 71.43% |
| P45 | 0.0294 | 0.0328 | 0.0333 | 0.0371 | 0.0284 | 0.0203 | 0.0263 | 0.0193 | 0.0211 | 0.0182 | 24.95% |
| P46 | 0.1608 | 0.1947 | 0.1446 | 0.1891 | 0.1747 | 0.1988 | 0.2706 | 0.2216 | 0.2424 | 0.2222 | 18.88% |
| P47 | 0.3311 | 0.3626 | 0.3063 | 0.3485 | 0.2913 | 0.2876 | 0.2886 | 0.2677 | 0.2259 | 0.0746 | 29.41% |
| P48 | 0.0202 | 0.0199 | 0.0143 | 0.0948 | 0.0193 | 0.0197 | 0.0196 | 0.0170 | 0.0167 | 0.0450 | 86.52% |
| P49 | 4.7166 | 5.0788 | 4.7450 | 6.0362 | 4.6871 | 5.3060 | 0.0042 | 5.2776 | 4.5929 | 3.4428 | 38.21% |
| P50 | 0.0109 | 0.3638 | 0.3540 | 0.4109 | 0.3236 | 0.3205 | 0.2948 | 0.2859 | 0.2371 | 0.1692 | 41.65% |
| P51 | 0.0772 | 0.0794 | 0.0768 | 0.1088 | 0.0665 | 0.0687 | 0.0595 | 0.0599 | 0.0573 | 0.0460 | 24.55% |
| P52 | 1.5887 | 1.8251 | 1.6828 | 2.0277 | 1.5861 | 1.6862 | 1.8476 | 1.7285 | 1.5471 | 1.2153 | 12.95% |
| P53 | 1.1882 | 0.3726 | 0.3821 | 0.4061 | 0.2821 | 0.3407 | 0.3619 | 0.3484 | 0.3332 | 0.2635 | 63.25% |
| P54 | 0.0993 | 0.2161 | 0.1311 | 0.1478 | 0.1073 | 0.1117 | 0.1172 | 0.1100 | 0.1134 | 0.0994 | 27.99% |
| P55 | 0.7732 | 0.8390 | 0.7138 | 0.9250 | 0.6054 | 0.7360 | 0.7145 | 0.6395 | 0.5735 | 0.3963 | 21.40% |
| P56 | 0.1557 | 0.1594 | 0.1338 | 0.1972 | 0.1940 | 0.1708 | 0.1750 | 0.1399 | 0.1790 | 0.1439 | 13.38% |
| P57 | 0.0989 | 0.1020 | 0.0986 | 0.1695 | 0.1040 | 0.1674 | 0.3199 | 0.1775 | 0.1192 | 0.1540 | 44.63% |
| P58 | 0.6627 | 0.7338 | 0.6786 | 0.9560 | 0.9087 | 0.9815 | 1.0607 | 1.1080 | 1.4908 | 1.2760 | 26.82% |
| P59 | 0.1007 | 0.1049 | 0.0744 | 0.1174 | 0.1031 | 0.0920 | 0.1038 | 0.0946 | 0.0946 | 0.0744 | 13.97% |
| P60 | 0.2304 | 0.2197 | 0.1728 | 0.2512 | 0.1903 | 0.2061 | 0.1592 | 0.2114 | 0.2385 | 0.3270 | 21.33% |
| P61 | 4.1004 | 4.6546 | 4.0480 | 5.3626 | 3.7408 | 4.2118 | 4.3333 | 4.0522 | 4.2451 | 0.0041 | 36.89% |
| P62 | 0.4594 | 0.4885 | 0.4595 | 0.6275 | 0.4199 | 0.4640 | 0.4731 | 0.4711 | 0.5021 | 0.3682 | 13.95% |
| P63 | 0.1284 | 0.1683 | 0.1677 | 0.2069 | 0.1347 | 0.2766 | 0.3360 | 0.2156 | 0.1920 | 0.0617 | 41.04% |
| P64 | 0.1077 | 0.1574 | 0.1085 | 0.1215 | 0.1432 | 0.1480 | 0.2014 | 0.1502 | 0.1846 | 0.1249 | 21.38% |
| P65 | 0.2406 | 0.2705 | 0.2238 | 0.3089 | 0.2841 | 0.3335 | 0.3281 | 0.3526 | 0.3465 | 0.3027 | 14.69% |
| P66 | 0.5155 | 0.5166 | 0.5474 | 0.6679 | 0.5241 | 0.5432 | 0.4701 | 0.4728 | 0.5512 | 0.3975 | 13.42% |
| P67 | 0.5034 | 0.5672 | 0.5305 | 0.7349 | 0.5650 | 0.5537 | 0.6072 | 0.5230 | 0.6478 | 0.4675 | 13.54% |
| P68 | 0.3269 | 0.1825 | 0.3882 | 0.4065 | 0.3401 | 0.3969 | 0.3756 | 0.5830 | 0.4537 | 0.3802 | 26.24% |
| P69 | 0.6047 | 0.5912 | 0.6303 | 0.7904 | 0.6595 | 0.7275 | 0.7075 | 0.6820 | 0.6379 | 0.5314 | 11.34% |
| P70 | 0.0080 | 0.0140 | 0.0116 | 0.0081 | 0.0094 | 0.0133 | 0.0070 | 0.0110 | 0.0099 | 0.0055 | 28.04% |
| P71 | 0.0321 | 0.0380 | 0.0319 | 0.0364 | 0.0331 | 0.0300 | 0.0338 | 0.0292 | 0.0322 | 0.0274 | 9.88% |
| P72 | 0.1828 | 0.1987 | 0.1581 | 0.2426 | 0.2108 | 0.2425 | 0.2173 | 0.2386 | 0.2775 | 0.2175 | 15.57% |
| P73 | 0.2906 | 0.2846 | 0.2595 | 0.3429 | 0.3206 | 0.3107 | 0.3088 | 0.2783 | 0.3051 | 0.2527 | 9.40% |
| P74 | 0.0847 | 0.1404 | 0.0811 | 0.1104 | 0.0988 | 0.1552 | 0.1677 | 0.0914 | 0.0862 | 0.0735 | 30.79% |
| P75 | 0.0340 | 0.0359 | 0.0312 | 0.0368 | 0.0237 | 0.0223 | 0.0175 | 0.0226 | 0.0243 | 0.0252 | 24.23% |
| P76 | 4.3669 | 4.8702 | 3.2623 | 5.8906 | 5.2085 | 5.3773 | 5.5371 | 5.1080 | 5.1887 | 4.2885 | 15.45% |
| P77 | 0.0057 | 0.0050 | 0.0068 | 0.0110 | 0.0099 | 0.0068 | 0.0077 | 0.0052 | 0.0079 | 0.0071 | 26.27% |
| P78 | 1.2175 | 1.3148 | 1.2641 | 1.5485 | 1.4525 | 1.3912 | 1.4859 | 1.4174 | 1.4311 | 1.0994 | 10.05% |
| P79 | 0.0821 | 0.0840 | 0.0849 | 0.0890 | 0.0744 | 0.0943 | 0.1019 | 0.2253 | 0.0740 | 0.0705 | 46.64% |
| P80 | 0.8443 | 0.8122 | 0.6273 | 0.9696 | 0.8890 | 0.9636 | 0.8550 | 0.9628 | 0.8278 | 0.6196 | 15.13% |
| P81 | 0.7769 | 0.7713 | 0.7625 | 0.8474 | 0.7442 | 0.6739 | 0.5133 | 0.5985 | 0.4510 | 0.2234 | 30.23% |
| P82 | 0.4693 | 0.3894 | 0.3797 | 0.4758 | 0.9868 | 1.0977 | 1.2409 | 0.6313 | 0.7295 | 1.7201 | 54.40% |
| P83 | 3.8882 | 4.2812 | 3.8543 | 4.8439 | 4.1375 | 3.9222 | 3.3828 | 3.7317 | 3.2840 | 2.0453 | 19.84% |
| P84 | 0.0799 | 0.0899 | 0.0830 | 0.0976 | 0.0832 | 0.0887 | 0.0917 | 0.1060 | 0.0971 | 0.0734 | 10.81% |
| P85 | 0.0680 | 0.3351 | 0.0690 | 0.0852 | 0.0999 | 0.1285 | 0.1054 | 0.0376 | 0.0245 | 0.1234 | 80.70% |
| P86 | 0.0256 | 0.0221 | 0.0263 | 0.0388 | 0.0321 | 0.0354 | 0.0354 | 0.0304 | 0.0382 | 0.0416 | 19.69% |
| P87 | 0.0651 | 0.0795 | 0.0664 | 0.0982 | 0.0837 | 0.0908 | 0.0692 | 0.0723 | 0.1115 | 0.0995 | 19.06% |
| P88 | 0.3080 | 0.3798 | 0.3385 | 0.4391 | 0.4255 | 0.4264 | 0.4450 | 0.4914 | 0.6289 | 0.6375 | 24.19% |
| P89 | 0.2612 | 0.2304 | 0.2276 | 0.2691 | 0.2565 | 0.2515 | 0.2274 | 0.2433 | 0.2376 | 0.1690 | 11.83% |
| P90 | 0.0214 | 0.0191 | 0.0054 | 0.0084 | 0.0064 | 0.0234 | 0.0072 | 0.0080 | 0.0102 | 0.0131 | 54.42% |
| P91 | 0.2936 | 0.3181 | 0.2897 | 0.3915 | 0.3433 | 0.3688 | 0.3892 | 0.3931 | 0.5003 | 0.5397 | 21.55% |
| P92 | 0.0129 | 0.0121 | 0.0132 | 0.0115 | 0.0108 | 0.0166 | 0.0124 | 0.0157 | 0.0160 | 0.0138 | 14.80% |
| P93 | 0.4369 | 0.3996 | 0.4401 | 0.0246 | 0.0250 | 0.0295 | 0.6397 | 0.6744 | 0.0680 | 0.8431 | 85.50% |
| P94 | 0.5389 | 0.7280 | 0.4887 | 0.5956 | 0.6503 | 0.6320 | 0.5965 | 0.8011 | 0.7954 | 1.0095 | 22.48% |
| P95 | 0.4981 | 0.4349 | 0.4217 | 0.5501 | 0.5750 | 0.5439 | 0.4454 | 0.4934 | 0.4822 | 0.3689 | 13.41% |

**Table S7**

Relative peak areas of common peaks in ten batches of JPYZXZ in negative ion mode.

| **ESI^−^** | **Samples** |  |  |  |  |  |  |  |  |  |  |
| --- | --- | --- | --- | --- | --- | --- | --- | --- | --- | --- | --- |
| **Peaks** | S1 | S2 | S3 | S4 | S5 | S6 | S7 | S8 | S9 | S10 | CV% |
| P1 | 0.7250 | 0.7428 | 0.6950 | 0.6262 | 1.5958 | 0.4502 | 0.4772 | 0.4706 | 0.4807 | 0.4583 | 51.39% |
| P2 | 0.3711 | 0.3607 | 0.3670 | 0.2817 | 0.1572 | 0.2399 | 0.2787 | 0.2533 | 0.2250 | 0.2425 | 25.23% |
| P3 | 0.9108 | 0.8275 | 0.8159 | 0.7033 | 0.4706 | 0.6476 | 0.7670 | 0.6284 | 0.6208 | 0.6138 | 18.60% |
| P4 | 1.6643 | 1.0549 | 1.0407 | 1.7003 | 1.6064 | 1.3814 | 0.6510 | 1.2386 | 1.1385 | 1.3057 | 25.65% |
| P5 | 0.1329 | 0.1440 | 0.1291 | 0.1391 | 0.0898 | 0.1312 | 0.1508 | 0.1343 | 0.1325 | 0.2769 | 33.35% |
| P6 | 1.0204 | 1.0091 | 0.9986 | 0.8650 | 0.3701 | 0.7672 | 1.0132 | 0.9407 | 0.7176 | 0.7170 | 24.51% |
| P7 | 0.2094 | 0.1944 | 0.1867 | 0.1734 | 0.0953 | 0.1562 | 0.1521 | 0.1526 | 0.1453 | 0.1524 | 19.62% |
| P8 | 0.1901 | 0.2636 | 0.2003 | 0.1822 | 0.1011 | 0.1859 | 0.2016 | 0.1865 | 0.1927 | 0.2453 | 21.95% |
| P9 | 0.1675 | 0.1601 | 0.1655 | 0.1496 | 0.0670 | 0.1318 | 0.1469 | 0.1476 | 0.1312 | 0.1347 | 20.61% |
| P10 | 0.5618 | 0.6244 | 0.4175 | 0.3343 | 0.1055 | 0.3002 | 0.2963 | 0.2427 | 0.2268 | 0.2683 | 46.49% |
| P11 | 1.0490 | 1.0737 | 0.9633 | 0.9035 | 0.5257 | 0.8589 | 0.9236 | 0.8220 | 0.7027 | 0.8503 | 18.65% |
| P12 | 0.1732 | 0.1721 | 0.1681 | 0.1643 | 0.1010 | 0.1500 | 0.1546 | 0.1638 | 0.1431 | 0.1601 | 13.72% |
| P13 | 0.2374 | 0.4491 | 0.4068 | 0.1975 | 0.1433 | 0.3354 | 0.3668 | 0.3568 | 0.4308 | 0.4391 | 32.09% |
| P14 | 0.2155 | 0.2058 | 0.1848 | 0.1940 | 0.1109 | 0.1746 | 0.1800 | 0.1785 | 0.1514 | 0.1624 | 16.86% |
| P15 | 0.5698 | 0.5815 | 0.5679 | 0.5253 | 0.3265 | 0.5233 | 0.5434 | 0.5164 | 0.5090 | 0.5093 | 13.94% |
| P16 | 0.4388 | 0.4637 | 0.4033 | 0.4073 | 0.1886 | 0.3855 | 0.4396 | 0.4234 | 0.3600 | 0.5644 | 23.24% |
| P17 | 0.5294 | 0.4807 | 0.4806 | 0.5126 | 0.2858 | 0.4080 | 0.5912 | 0.4195 | 0.4728 | 0.3143 | 21.06% |
| P18 | 0.5095 | 0.6491 | 0.5229 | 0.6894 | 0.2198 | 0.3983 | 0.4123 | 0.4798 | 0.4915 | 0.4565 | 27.15% |
| P19 | 0.1915 | 0.2165 | 0.1141 | 0.2837 | 0.1048 | 0.0985 | 0.2179 | 0.1173 | 0.1018 | 0.1109 | 42.39% |
| P20 | 0.3048 | 0.3099 | 0.3175 | 0.2892 | 0.1400 | 0.2957 | 0.3249 | 0.3077 | 0.2788 | 0.3036 | 18.59% |
| P21 | 0.7762 | 0.6664 | 0.6100 | 0.5824 | 0.2845 | 0.5513 | 0.5987 | 0.4830 | 0.4785 | 0.5541 | 23.20% |
| P22 | 2.3545 | 2.2358 | 1.9321 | 1.8476 | 0.0008 | 1.9206 | 1.6418 | 2.1318 | 1.4153 | 0.0018 | 55.53% |
| P23 | 0.0049 | 0.0070 | 0.0080 | 0.0070 | 0.0022 | 0.0064 | 0.0053 | 0.0058 | 0.0055 | 0.0067 | 27.22% |
| P24 | 1.1947 | 1.0786 | 1.0963 | 1.0747 | 0.7418 | 0.9649 | 0.9577 | 0.9808 | 1.0024 | 1.0940 | 12.00% |
| P25 | 0.2344 | 0.2128 | 0.2842 | 0.2047 | 0.1076 | 0.1945 | 0.2055 | 0.1971 | 0.1699 | 0.1764 | 22.83% |
| P26 | 0.2903 | 0.2766 | 0.2817 | 0.2663 | 0.1673 | 0.2372 | 0.2541 | 0.2543 | 0.2565 | 0.2413 | 13.65% |
| P27 | 0.7519 | 0.6874 | 0.6729 | 0.6464 | 0.4419 | 0.6036 | 0.5739 | 0.6302 | 0.5910 | 0.6411 | 13.17% |
| P28 | 0.7729 | 0.6825 | 0.6550 | 0.6488 | 0.3956 | 0.6057 | 0.6270 | 0.6143 | 0.6009 | 0.6207 | 15.18% |
| P29 | 2.3270 | 2.2050 | 2.0424 | 2.0353 | 1.4456 | 1.8929 | 1.9937 | 1.9779 | 1.8799 | 2.3026 | 12.59% |
| P30 | 0.1706 | 0.1536 | 0.1561 | 0.1346 | 0.0739 | 0.1237 | 0.1427 | 0.1217 | 0.1190 | 0.1413 | 20.00% |
| P31 | 0.0209 | 0.0171 | 0.0142 | 0.0157 | 0.0130 | 0.0184 | 0.0122 | 0.0218 | 0.0114 | 0.0224 | 24.34% |
| P32 | 1.1350 | 1.0994 | 0.8347 | 1.0046 | 0.8492 | 0.8953 | 0.9146 | 0.8395 | 1.0236 | 1.0264 | 11.45% |
| P33 | 0.3306 | 0.3433 | 0.2805 | 0.3140 | 0.2250 | 0.3081 | 0.3035 | 0.3050 | 0.3032 | 0.3044 | 10.55% |
| P34 | 0.3919 | 0.3934 | 0.3254 | 0.3277 | 0.2349 | 0.3494 | 0.3244 | 0.3550 | 0.2509 | 0.3475 | 15.81% |
| P35 | 0.4344 | 0.4216 | 0.4153 | 0.3678 | 0.1915 | 0.3567 | 0.3772 | 0.3731 | 0.3302 | 0.4161 | 19.11% |
| P36 | 3.3250 | 3.1698 | 3.1324 | 3.0991 | 1.7460 | 2.6278 | 3.0290 | 2.8076 | 2.5502 | 2.7307 | 16.14% |
| P37 | 0.2843 | 0.2693 | 0.2803 | 0.2622 | 0.1751 | 0.2559 | 0.2793 | 0.2733 | 0.2383 | 0.2910 | 12.96% |
| P38 | 0.2004 | 0.1850 | 0.1804 | 0.1548 | 0.1127 | 0.1517 | 0.1513 | 0.1454 | 0.1281 | 0.1495 | 16.92% |
| P39 | 0.1758 | 0.1709 | 0.1731 | 0.1657 | 0.0791 | 0.1606 | 0.1652 | 0.1677 | 0.1464 | 0.1643 | 18.16% |
| P40 | 0.5611 | 0.5217 | 0.4592 | 0.5159 | 0.3267 | 0.4786 | 0.4909 | 0.5039 | 0.4719 | 0.4393 | 13.24% |
| P41 | 0.2373 | 0.2201 | 0.1828 | 0.1947 | 0.1295 | 0.1887 | 0.2023 | 0.1766 | 0.1743 | 0.1830 | 15.27% |
| P42 | 0.7357 | 0.6833 | 0.6232 | 0.6254 | 0.4333 | 0.6170 | 0.6329 | 0.6394 | 0.5728 | 0.6879 | 13.00% |
| P43 | 0.0232 | 0.0252 | 0.0195 | 0.0216 | 0.0133 | 0.0199 | 0.0189 | 0.0209 | 0.0193 | 0.0181 | 15.93% |
| P44 | 7.3934 | 6.9597 | 6.8534 | 6.4213 | 5.0198 | 6.3739 | 6.4072 | 5.9391 | 6.0921 | 6.2906 | 10.09% |
| P45 | 0.3962 | 0.3747 | 0.3412 | 0.3175 | 0.2261 | 0.2834 | 0.3198 | 0.2693 | 0.2721 | 0.2749 | 17.05% |
| P46 | 0.2667 | 0.2441 | 0.2384 | 0.2363 | 0.1377 | 0.2226 | 0.2337 | 0.2177 | 0.2476 | 0.2347 | 15.12% |
| P47 | 0.0802 | 0.0785 | 0.0613 | 0.0603 | 0.0435 | 0.0661 | 0.0534 | 0.0682 | 0.0521 | 0.0765 | 19.08% |
| P48 | 0.0187 | 0.0201 | 0.0201 | 0.0147 | 0.0111 | 0.0143 | 0.0134 | 0.0147 | 0.0116 | 0.0151 | 20.99% |
| P49 | 0.3188 | 0.3031 | 0.2928 | 0.2899 | 0.1288 | 0.2820 | 0.2703 | 0.3206 | 0.2834 | 0.3102 | 19.86% |
| P50 | 0.3801 | 0.3745 | 0.2924 | 0.2942 | 0.2270 | 0.3102 | 0.2996 | 0.3044 | 0.2818 | 0.3329 | 14.43% |
| P51 | 0.2630 | 0.2358 | 0.2990 | 0.2385 | 0.1414 | 0.2254 | 0.2387 | 0.2397 | 0.2258 | 0.3143 | 19.29% |
| P52 | 0.0040 | 0.0036 | 0.0043 | 0.0037 | 0.0010 | 0.0032 | 0.0043 | 0.0047 | 0.0045 | 0.0047 | 28.67% |
| P53 | 0.1377 | 0.1378 | 0.1363 | 0.1487 | 0.0694 | 0.1368 | 0.1479 | 0.1548 | 0.1241 | 0.1395 | 18.00% |
| P54 | 1.3697 | 1.3233 | 1.1863 | 1.1897 | 0.7335 | 1.1355 | 1.1572 | 1.1555 | 1.0593 | 1.1590 | 14.91% |
| P55 | 0.0122 | 0.0118 | 0.0090 | 0.0093 | 0.0102 | 0.0104 | 0.0073 | 0.0075 | 0.0074 | 0.0086 | 19.04% |
| P56 | 0.0321 | 0.0349 | 0.0278 | 0.0273 | 0.0267 | 0.0328 | 0.0277 | 0.0236 | 0.0264 | 0.0426 | 18.39% |
| P57 | 0.0700 | 0.0714 | 0.0600 | 0.0710 | 0.0395 | 0.0631 | 0.0646 | 0.0628 | 0.0601 | 0.0821 | 17.14% |
| P58 | 0.0166 | 0.0160 | 0.0154 | 0.0142 | 0.0078 | 0.0137 | 0.0132 | 0.0135 | 0.0150 | 0.0141 | 17.31% |
| P59 | 0.5738 | 0.5041 | 0.4986 | 0.4937 | 0.2452 | 0.4526 | 0.5432 | 0.4525 | 0.4177 | 0.5042 | 19.31% |
| P60 | 0.8234 | 0.7811 | 0.8142 | 0.8309 | 0.4876 | 0.8016 | 0.8573 | 0.8380 | 0.7103 | 0.7786 | 14.00% |
| P61 | 0.1823 | 0.1844 | 0.1743 | 0.1803 | 0.0801 | 0.1743 | 0.1887 | 0.1923 | 0.1740 | 0.1777 | 19.02% |
| P62 | 0.1519 | 0.1543 | 0.1463 | 0.1458 | 0.0655 | 0.1484 | 0.1672 | 0.1614 | 0.1382 | 0.1488 | 19.86% |
| P63 | 0.2887 | 0.2905 | 0.2477 | 0.2633 | 0.1207 | 0.2597 | 0.2847 | 0.2729 | 0.2251 | 0.2332 | 20.21% |
| P64 | 0.2096 | 0.1804 | 0.1727 | 0.1863 | 0.0952 | 0.1820 | 0.2020 | 0.1761 | 0.2011 | 0.1703 | 17.94% |
| P65 | 0.0051 | 0.0046 | 0.0037 | 0.0049 | 0.0022 | 0.0042 | 0.0031 | 0.0032 | 0.0034 | 0.0046 | 24.24% |
| P66 | 5.1683 | 4.7097 | 4.4870 | 4.4485 | 1.9437 | 4.4571 | 4.1509 | 4.9786 | 4.2620 | 4.8801 | 20.79% |
| P67 | 0.0515 | 0.0483 | 0.0422 | 0.0429 | 0.0262 | 0.0389 | 0.0453 | 0.0426 | 0.0440 | 0.0387 | 16.19% |
| P68 | 0.0044 | 0.0055 | 0.0044 | 0.0044 | 0.0025 | 0.0051 | 0.0040 | 0.0037 | 0.0043 | 0.0051 | 19.62% |
| P69 | 0.2868 | 0.2551 | 0.2573 | 0.1323 | 0.1168 | 0.2653 | 0.2918 | 0.2720 | 0.1371 | 0.2635 | 30.52% |
| P70 | 2.0986 | 3.8471 | 2.0552 | 0.8923 | 0.8357 | 1.8481 | 2.1329 | 2.1083 | 0.8174 | 2.0968 | 48.10% |
| P71 | 0.0064 | 0.0057 | 0.0067 | 0.0029 | 0.0025 | 0.0057 | 0.0062 | 0.0047 | 0.0022 | 0.0045 | 35.55% |
| P72 | 0.0075 | 0.0073 | 0.0078 | 0.0037 | 0.0035 | 0.0066 | 0.0075 | 0.0071 | 0.0034 | 0.0073 | 29.58% |
| P73 | 0.1028 | 0.0997 | 0.1029 | 0.0454 | 0.0498 | 0.0883 | 0.0962 | 0.0879 | 0.0478 | 0.0911 | 29.27% |
| P74 | 0.0031 | 0.0029 | 0.0021 | 0.0009 | 0.0007 | 0.0020 | 0.0016 | 0.0018 | 0.0010 | 0.0019 | 44.60% |
| P75 | 0.0071 | 0.0065 | 0.0055 | 0.0031 | 0.0027 | 0.0053 | 0.0056 | 0.0048 | 0.0034 | 0.0052 | 29.73% |
| P76 | 0.0048 | 0.0050 | 0.0048 | 0.0029 | 0.0016 | 0.0046 | 0.0040 | 0.0039 | 0.0024 | 0.0043 | 29.84% |
| P77 | 0.2128 | 0.2204 | 0.2148 | 0.1281 | 0.0874 | 0.2347 | 0.2620 | 0.2515 | 0.1308 | 0.2373 | 30.35% |
| P78 | 0.1135 | 0.1252 | 0.2506 | 0.2368 | 0.0656 | 0.2017 | 0.1421 | 0.2200 | 0.2790 | 0.3001 | 40.29% |
| P79 | 0.0058 | 0.0067 | 0.0143 | 0.0123 | 0.0030 | 0.0099 | 0.0057 | 0.0069 | 0.0117 | 0.0063 | 43.15% |
| P80 | 0.0049 | 0.0048 | 0.0115 | 0.0085 | 0.0019 | 0.0047 | 0.0049 | 0.0030 | 0.0091 | 0.0037 | 52.93% |
| P81 | 0.3245 | 0.0355 | 0.5872 | 0.4487 | 0.1678 | 0.2929 | 0.0481 | 0.2138 | 0.4581 | 0.2844 | 62.41% |
| P82 | 0.0089 | 0.0089 | 0.0087 | 0.0086 | 0.0085 | 0.0086 | 0.0088 | 0.0084 | 0.0087 | 0.0089 | 1.84% |
| P83 | 0.0079 | 0.0073 | 0.0144 | 0.0073 | 0.0027 | 0.0068 | 0.0072 | 0.0066 | 0.0200 | 0.0091 | 54.04% |
| P84 | 0.0097 | 0.0103 | 0.0142 | 0.0090 | 0.0033 | 0.0081 | 0.0087 | 0.0087 | 0.0126 | 0.0081 | 31.41% |
| P85 | 0.0042 | 0.0052 | 0.0063 | 0.0052 | 0.0013 | 0.0054 | 0.0056 | 0.0054 | 0.0051 | 0.0045 | 27.96% |
| P86 | 0.0722 | 0.0655 | 0.0603 | 0.0510 | 0.0248 | 0.0436 | 0.0430 | 0.0434 | 0.0408 | 0.0397 | 29.00% |
| P87 | 0.3701 | 0.3928 | 0.3521 | 0.2230 | 0.2265 | 0.2705 | 0.2141 | 0.2467 | 0.2498 | 0.2284 | 24.38% |
| P88 | 0.0318 | 0.0269 | 0.0206 | 0.0208 | 0.0093 | 0.0196 | 0.0190 | 0.0194 | 0.0134 | 0.0173 | 31.61% |
| P89 | 4.4155 | 6.4552 | 6.1888 | 5.4199 | 5.2239 | 5.3605 | 5.3412 | 5.1392 | 3.7222 | 5.3581 | 14.77% |

**Table S8. The metabolites in JPYZXZ identified by UPLC-Orbitrap-MS/MS in positive ion mode.**

| Peaks | tR (min) | Identification | Molecular Formula | m/z (Da) | Score (Final) | Adducts |
| --- | --- | --- | --- | --- | --- | --- |
| P1 | 0.6321 | Gentianaine | C6H7NO3 | 142.0527 | 0.3961 | M+H |
| P2 | 0.9997 | Homostachydrine | C8H15NO2 | 158.1172 | 0.6005 | M+H |
| P3 | 1.2489 | Arecoline | C8H13NO2 | 156.1017 | 0.4643 | M+H |
| P4 | 2.2368 | Protoanemonin | C5H4O2 | 97.0288 | 0.6306 | M+H |
| P5 | 2.4476 | Kojic acid | C6H6O4 | 143.0337 | 0.6737 | M+H |
| P6 | 2.6591 | Vanillylamine | C8H11NO2 | 154.0860 | 0.5630 | M+H |
| P7 | 2.8674 | 5-Aminopentanal | C5H11NO | 102.0916 | 0.6032 | M+H |
| P8 | 2.9071 | Gentisate aldehyde | C7H6O3 | 139.0388 | 0.3953 | M+H |
| P9 | 3.2365 | Retronecine | C8H13NO2 | 156.1017 | 0.3104 | M+H |
| P10 | 3.4241 | Juglone | C10H6O3 | 175.0388 | 0.5986 | M+H |
| P11 | 3.6309 | chlorogenic acid | C16H18O9 | 355.1015 | 0.7704 | M+H |
| P12 | 4.3930 | Licoagroside B | C18H24O12 | 433.1331 | 0.9400 | M+H |
| P13 | 4.4315 | Riboflavin B2 | C17H20N4O6 | 377.1447 | 0.6531 | M+H |
| P14 | 4.7833 | Delphinidin 3-O-sophoroside | C27H31O17 | 627.1539 | 0.6664 | M+H |
| P15 | 4.9317 | Corymboside | C26H28O14 | 565.1540 | 0.8252 | M+H |
| P16 | 4.9547 | Dihydrogenistein | C15H12O5 | 273.0752 | 0.6235 | M+H |
| P17 | 5.3148 | Naringenin | C15H12O5 | 273.0752 | 0.7590 | M+H |
| P18 | 5.4256 | Rutin | C27H30O16 | 611.1592 | 0.7891 | M+H |
| P19 | 5.5620 | Quercetin 3-D-galactoside | C21H20O12 | 465.1017 | 0.7009 | M+H |
| P20 | 5.9462 | Paeonilactone C | C17H18O6 | 319.1168 | 0.8018 | M+H |
| P21 | 5.9861 | Kaempferol 3-O-β-rutinoside | C27H30O15 | 595.1642 | 0.7902 | M+H |
| P22 | 6.1051 | 1,3-Dicaffeoylquinic acid | C25H24O12 | 517.1330 | 0.8364 | M+H |
| P23 | 6.1580 | Kaempferol -3-glucoside | C21H20O11 | 449.1067 | 0.7402 | M+H |
| P24 | 6.2839 | Naringin | C27H32O14 | 581.1851 | 0.6556 | M+H |
| P25 | 6.3009 | loliolide | C11H16O3 | 197.1170 | 0.6699 | M+H |
| P26 | 6.4493 | p-Methoxycinnamaldehyde | C10H10O2 | 163.0751 | 0.5918 | M+H |
| P27 | 6.4789 | Isorhamnetin-3-O-rutinoside | C28H32O16 | 625.1743 | 0.8991 | M+H |
| P28 | 6.4826 | Peonidin 3-O-glucoside | C22H23O11 | 463.1224 | 0.7754 | M+H |
| P29 | 6.5812 | Malonylglycitin | C25H24O13 | 533.1278 | 0.7565 | M+H |
| P30 | 6.7258 | sorbitol | C6H14O6 | 183.0650 | 0.7249 | M+H |
| P31 | 6.7332 | Hesperidin | C28H34O15 | 611.1955 | 0.8609 | M+H |
| P32 | 6.8804 | Sarisan | C11H12O3 | 193.0857 | 0.7883 | M+H |
| P33 | 7.0812 | Albiflorin | C23H28O11 | 481.1693 | 0.7829 | M+H |
| P34 | 7.1328 | Damascenone | C13H18O | 191.1426 | 0.7134 | M+H |
| P35 | 7.1650 | Isoliquiritin | C21H22O9 | 419.1326 | 0.5926 | M+H |
| P36 | 7.2836 | Ononin | C22H22O9 | 431.1327 | 0.8868 | M+H |
| P37 | 7.2843 | Formononetin | C16H12O4 | 269.0800 | 0.7261 | M+H |
| P38 | 7.3039 | Acetyleugenol | C12H14O3 | 207.1012 | 0.7146 | M+H |
| P39 | 7.4734 | Isoliquiritigenin | C15H12O4 | 257.0802 | 0.8193 | M+H |
| P40 | 7.5489 | Quercetin | C15H10O7 | 303.0492 | 0.5625 | M+H |
| P41 | 7.9232 | Exoticin | C23H26O10 | 463.1586 | 0.6584 | M+H |
| P42 | 7.9806 | Megastigmatrienone | C13H18O | 191.1427 | 0.6110 | M+H |
| P43 | 8.0026 | Canin | C15H18O5 | 279.1221 | 0.6121 | M+H |
| P44 | 8.0710 | Pinocembrin | C15H12O4 | 257.0804 | 0.7020 | M+H |
| P45 | 8.1230 | Formononetin 7-O-glucoside-6''-O-malonate | C25H24O12 | 517.1329 | 0.8022 | M+H |
| P46 | 8.1959 | 3-Dimethylallyl-4-hydroxybenzaldehyde | C12H14O2 | 191.1064 | 0.6773 | M+H |
| P47 | 8.5352 | 3,4-Methylenesebacic acid | C12H18O4 | 227.1273 | 0.6133 | M+H |
| P48 | 8.6030 | Isosakuranetin | C16H14O5 | 287.0906 | 0.6615 | M+H |
| P49 | 8.9729 | Curcumenone | C15H22O2 | 235.1689 | 0.7602 | M+H |
| P50 | 9.0084 | Hesperetin | C16H14O6 | 303.0857 | 0.6440 | M+H |
| P51 | 9.0260 | Formononetin 7-O-(6''-acetylglcoside) | C24H24O10 | 473.1432 | 0.7982 | M+H |
| P52 | 9.0653 | α-Curcumene | C15H22 | 203.1792 | 0.6806 | M+H |
| P53 | 9.1630 | Linderalactone | C15H16O3 | 245.1168 | 0.6921 | M+H |
| P54 | 9.3869 | Dehydrozingerone | C11H12O3 | 193.0857 | 0.6152 | M+H |
| P55 | 9.4524 | Dehydrovomifoliol | C13H18O3 | 223.1325 | 0.6744 | M+H |
| P56 | 9.5020 | Daidzein | C15H10O4 | 255.0647 | 0.5589 | M+H |
| P57 | 9.5077 | Cyperotundone | C15H22O | 218.1617 | 0.6889 | M+H |
| P58 | 9.5170 | 4-Methyl-2-phenyl-2-pentenal | C12H14O | 175.1115 | 0.6153 | M+H |
| P59 | 9.5800 | Biochanin A | C16H12O5 | 285.0751 | 0.6488 | M+H |
| P60 | 9.6977 | Curdione | C15H24O2 | 237.1844 | 0.6076 | M+H |
| P61 | 9.7569 | Portulacaxanthin III | C11H12N2O6 | 269.0804 | 0.6179 | M+H |
| P62 | 9.7586 | Tangeretin | C20H20O7 | 373.1273 | 0.8911 | M+H |
| P63 | 9.8169 | 3-Dimethylallyl-4-hydroxybenzoate | C12H14O3 | 207.1013 | 0.6235 | M+H |
| P64 | 9.8333 | Perillyl aldehyde | C10H14O | 151.1115 | 0.4171 | M+H |
| P65 | 9.9078 | Chavicol | C9H10O | 135.0803 | 0.5195 | M+H |
| P66 | 10.3389 | Sinensetin | C20H20O7 | 373.1273 | 0.8153 | M+H |
| P67 | 10.5039 | 4',5,6,7-Tetramethoxyflavone | C19H18O6 | 343.1168 | 0.6783 | M+H |
| P68 | 10.5656 | β-Lonone | C13H20O | 175.1478 | 0.6293 | M+H-H2O |
| P69 | 10.5706 | Isozaluzanin C | C15H18O3 | 247.1324 | 0.6296 | M+H |
| P70 | 10.6571 | Medicarpin | C16H14O4 | 271.0957 | 0.5546 | M+H |
| P71 | 10.6778 | Centratherin | C20H22O7 | 375.1429 | 0.6595 | M+H |
| P72 | 10.8096 | Safranal | C10H14O | 151.1114 | 0.3860 | M+H |
| P73 | 11.0081 | Cedrelopsin | C15H16O4 | 261.1116 | 0.8847 | M+H |
| P74 | 11.1083 | Encelin | C15H16O3 | 245.1168 | 0.6074 | M+H |
| P75 | 11.2024 | Glycyrrhetinate | C30H46O4 | 471.3459 | 0.7548 | M+H |
| P76 | 11.2262 | Alantolactone | C15H20O2 | 233.1532 | 0.6565 | M+H |
| P77 | 11.2631 | Soyaspongenol C | C30H48O2 | 441.3714 | 0.5851 | M+H |
| P78 | 11.6902 | Isoalantolactone | C15H20O2 | 233.1532 | 0.8020 | M+H |
| P79 | 11.8068 | 2-(-)-Carvone | C10H14O | 151.1115 | 0.4345 | M+H |
| P80 | 11.9614 | α-Sinensal | C15H22O | 219.1740 | 0.7776 | M+H |
| P81 | 12.0377 | Parthenolide | C15H20O3 | 249.1479 | 0.7457 | M+H |
| P82 | 12.0574 | Guaiazulene | C15H18 | 199.1478 | 0.7379 | M+H |
| P83 | 12.0928 | Zederone | C15H18O3 | 247.1324 | 0.8815 | M+H |
| P84 | 12.4866 | Nobiletin | C21H22O8 | 403.1377 | 0.8806 | M+H |
| P85 | 12.5619 | Boschniakine | C10H11NO | 162.0910 | 0.6213 | M+H |
| P86 | 12.5998 | 6β,7β-Dihydroxykaurenoic acid | C20H30O4 | 335.2186 | 0.6945 | M+H |
| P87 | 12.6751 | Niranthin | C24H32O7 | 415.2105 | 0.9605 | M+H-H2O |
| P88 | 12.6791 | Farfugin A | C15H18O | 215.1427 | 0.5964 | M+H |
| P89 | 12.7243 | Solavetivol | C15H24O | 221.1896 | 0.7410 | M+H |
| P90 | 13.1836 | Sclareolide | C16H26O2 | 251.2000 | 0.6084 | M+H |
| P91 | 13.3554 | 1,2-Cyclohexanediol, 1-methyl-4-(1-methylethenyl)-, 2-benzoate, (1S,2S,4R)- | C17H22O3 | 257.1531 | 0.9484 | M+H-H2O |
| P92 | 13.4120 | Betulonic acid | C30H46O3 | 455.3507 | 0.5883 | M+H |
| P93 | 13.4763 | Chamazulene | C14H16 | 185.1321 | 0.6310 | M+H |
| P94 | 14.5315 | 5,6-Dihydrochamazulene | C14H18 | 187.1479 | 0.7164 | M+H |
| P95 | 14.8428 | Drimenin | C15H22O2 | 235.1688 | 0.7786 | M+H |

**Table S9. The metabolites in JPYZXZ identified by UPLC-Orbitrap-MS/MS in negative ion mode.**

| Peaks | tR (min) | Identification | Molecular Formula | m/z (Da) | Score (Final) | Adducts |
| --- | --- | --- | --- | --- | --- | --- |
| P1 | 1.0872 | Vanillic acid | C8H8O4 | 167.0339 | 0.7152 | M-H |
| P2 | 1.1955 | Dl-P-Hydroxyphenyl lactic acid | C9H10O4 | 181.0498 | 0.6385 | M-H |
| P3 | 1.3532 | Guaiacol | C7H8O2 | 123.0438 | 0.7344 | M-H |
| P4 | 1.3808 | Azelaic acid | C9H16O4 | 187.0967 | 0.7761 | M-H |
| P5 | 1.4482 | Kojic acid | C6H6O4 | 141.0180 | 0.7882 | M-H |
| P6 | 1.9023 | 6-Hydroxyhexanoic acid | C6H12O3 | 131.0702 | 0.6856 | M-H |
| P7 | 2.0777 | Mitoxantrone | C22H28N4O6 | 443.1926 | 0.6823 | M-H |
| P8 | 2.4018 | 2,3-Butanediol glucoside | C10H20O7 | 251.1131 | 0.6384 | M-H |
| P9 | 2.7691 | Cynaratriol | C15H22O5 | 281.1392 | 0.6180 | M-H |
| P10 | 3.8192 | Avenanthramide E | C17H15NO5 | 312.0948 | 0.9220 | M-H |
| P11 | 3.8952 | 3-Dehydroshikimic acid | C7H8O5 | 153.0183 | 0.6327 | M-H2O-H |
| P12 | 4.0005 | Vanillin | C8H8O3 | 151.0391 | 0.6328 | M-H |
| P13 | 4.1436 | (+)-threo-2-Amino-3,4-dihydroxybutanoic acid | C4H9NO4 | 134.0459 | 0.7611 | M-H |
| P14 | 4.6769 | Vicenin-2 | C27H30O15 | 593.1510 | 0.6758 | M-H |
| P15 | 4.7087 | Theviridoside | C17H24O11 | 403.1250 | 0.6610 | M-H |
| P16 | 5.3975 | Artabsinolide D | C15H22O5 | 281.1395 | 0.7149 | M-H |
| P17 | 5.5840 | Genipin | C11H14O5 | 225.0766 | 0.9056 | M-H |
| P18 | 5.7272 | cis-3-Hexenyl lactate | C9H16O3 | 171.1017 | 0.8433 | M-H |
| P19 | 5.8831 | 7-hydroxy-coumarin | | 161.0232 | 0.6644 | M-H |
| P20 | 5.9152 | Vulgarolide | C15H20O5 | 279.1233 | 0.7218 | M-H |
| P21 | 6.0299 | Sagerinic acid | C36H32O16 | 719.1622 | 0.7086 | M-H |
| P22 | 6.1482 | Liquiritin | C21H22O9 | 417.1184 | 0.7090 | M-H |
| P23 | 6.2718 | Syringin | C17H24O9 | 743.2747 | 0.6555 | 2M-H |
| P24 | 6.3888 | Isochlorogenic acid b | C25H24O12 | 515.1182 | 0.7130 | M-H |
| P25 | 6.3893 | 4,8-Dihydroxy-6-(hydroxymethyl)-6,8-dimethyl-4,4a,5,6,7,7a,8,9-octahydroazuleno[5,6-c]furan-1(3H)-one | C15H22O5 | 281.1392 | 0.6168 | M-H |
| P26 | 6.4521 | Quercetin 3-β-D-glucoside | C21H20O12 | 463.0888 | 0.7971 | M-H |
| P27 | 6.5860 | 4,5-Di-O-caffeoylquinic acid | C25H24O12 | 515.1180 | 0.7564 | M-H |
| P28 | 6.6187 | Rutin | C27H30O16 | 609.1444 | 0.7852 | M-H |
| P29 | 7.2471 | Albiflorin | C23H28O11 | 479.1555 | 0.9501 | M-H |
| P30 | 7.3722 | 5-Hydroxyindoleacetaldehyde | C10H9NO2 | 174.0553 | 0.6891 | M-H |
| P31 | 7.6439 | Licoricesaponin G2 | C42H62O17 | 837.3897 | 0.4677 | M-H |
| P32 | 7.7460 | Liquiritigenin | C15H12O4 | 255.0661 | 0.8714 | M-H |
| P33 | 8.0091 | Dihydrogenistein | C15H12O5 | 271.0608 | 0.6296 | M-H |
| P34 | 8.0487 | Neoliquiritin 2''-apioside | C26H30O13 | 549.1608 | 0.8048 | M-H |
| P35 | 8.0746 | 4-Hydroxyquinoline | C9H7NO | 144.0442 | 0.9561 | M-H |
| P36 | 8.1639 | 3,4,5-Trimethoxycinnamic acid | C12H14O5 | 237.0762 | 0.7048 | M-H |
| P37 | 8.4162 | Genkwanin | C16H12O5 | 284.0640 | 0.8215 | M-H |
| P38 | 8.4162 | Trifolirhizin | C22H22O10 | 445.1146 | 0.6738 | M-H |
| P39 | 8.7418 | Umbellifolide | C15H20O4 | 263.1288 | 0.6998 | M-H |
| P40 | 8.8022 | Hesperetin | C16H14O6 | 301.0716 | 0.9255 | M-H |
| P41 | 8.9476 | Daidzein | C15H10O4 | 253.0506 | 0.6766 | M-H |
| P42 | 9.2336 | Wogonin | C16H12O5 | 284.0640 | 0.9650 | M-H |
| P43 | 9.2776 | Licorice glycoside C1 | C36H38O16 | 725.2087 | 0.6165 | M-H |
| P44 | 9.4566 | Epihesperidin | C28H34O15 | 609.1827 | 0.8814 | M-H |
| P45 | 9.5255 | Parthenin | C15H18O4 | 261.1133 | 0.7192 | M-H |
| P46 | 9.8405 | Secoisolariciresinol | C20H26O6 | 361.1654 | 0.6373 | M-H |
| P47 | 10.2668 | Uralsaponin B | C42H62O16 | 821.3959 | 0.5406 | M-H |
| P48 | 10.3422 | Licoricesaponin E2 | C42H60O16 | 819.3790 | 0.5131 | M-H |
| P49 | 10.3536 | Ilicic acid | C15H24O3 | 251.1649 | 0.8001 | M-H |
| P50 | 10.4869 | Glycyrrhizic acid | C42H62O16 | 821.3956 | 0.8053 | M-H |
| P51 | 10.4988 | Glycitein | C16H12O5 | 283.0608 | 0.8490 | M-H |
| P52 | 10.8786 | β-vulgaroside IV | C41H62O15 | 793.4031 | 0.6706 | M-H |
| P53 | 10.9700 | Jasmolone | C11H16O2 | 179.1069 | 0.5762 | M-H |
| P54 | 11.0138 | tiliroside | C30H26O13 | 593.1882 | 0.6699 | M-H |
| P55 | 11.0578 | 28-Glucosyl-3b-hydroxy-12-oleanene-30-methoxy-28-oic acid 3-[arabinosyl-(1->3)-glucuronide] | C48H74O20 | 969.4703 | 0.5651 | M-H |
| P56 | 11.1469 | Oleragenoside | C42H64O16 | 823.4097 | 0.5181 | M-H |
| P57 | 11.1850 | Licoricesaponin A3 | C48H72O21 | 983.4477 | 0.4410 | M-H |
| P58 | 11.2516 | Centellasaponin B | C42H68O16 | 827.4411 | 0.4903 | M-H |
| P59 | 11.4831 | Eupolauridine | C14H8N2 | 203.0709 | 0.6381 | M-H |
| P60 | 11.5317 | 4-Hydroxydehydromyoporone | C15H20O4 | 263.1288 | 0.6775 | M-H |
| P61 | 12.2132 | Istanbulin A | C15H20O4 | 263.1288 | 0.6348 | M-H |
| P62 | 12.3475 | Ipomeabisfuran | C15H18O3 | 245.1182 | 0.6858 | M-H |
| P63 | 12.3917 | 2'-Hydroxyflavone | C15H10O3 | 237.0554 | 0.9496 | M-H |
| P64 | 12.3928 | Isolicoflavonol | C20H18O6 | 353.1034 | 0.7820 | M-H |
| P65 | 12.5068 | Dehydrosoyasaponin I | C48H76O18 | 939.4952 | 0.6983 | M-H |
| P66 | 12.5181 | Furanogermenone | C15H20O2 | 231.1386 | 0.8521 | M-H |
| P67 | 12.5757 | Licoricesaponin C2 | C42H62O15 | 805.4017 | 0.4839 | M-H |
| P68 | 12.8360 | Chikusetsusaponin V | C48H76O19 | 955.4887 | 0.7709 | M-H |
| P69 | 13.6869 | Semilicoisoflavone B | C20H16O6 | 351.0875 | 0.8027 | M-H |
| P70 | 13.7793 | Quadrone | C15H20O3 | 247.1334 | 0.6354 | M-H |
| P71 | 13.8886 | Araliasaponin I | C47H76O18 | 927.4935 | 0.6531 | M-H |
| P72 | 13.9026 | Soyasaponin II | C47H76O17 | 911.5017 | 0.6082 | M-H |
| P73 | 13.9287 | Arvensoside A | C48H78O18 | 941.5121 | 0.4252 | M-H |
| P74 | 14.0542 | 3-Glu-28-Glu-Bayogenin | C42H68O15 | 857.4539 | 0.9997 | M-H |
| P75 | 14.1225 | Soyasaponin III | C42H68O14 | 795.4530 | 0.7370 | M-H |
| P76 | 14.2296 | Goyaglycoside h | C42H70O15 | 813.4638 | 0.6864 | M-H |
| P77 | 14.4229 | Curdione | C15H24O2 | 235.1698 | 0.6652 | M-H |
| P78 | 15.0925 | Caryophyllene epoxide | C15H24O | 219.1747 | 0.7129 | M-H |
| P79 | 15.1610 | Hoduloside VI | C41H68O14 | 783.4519 | 0.5553 | M-H |
| P80 | 15.2415 | Vinaginsenoside R11 | C41H70O14 | 785.4676 | 0.9030 | M-H |
| P81 | 15.3811 | 3-Hydroxy-3',4'-Dimethoxyflavone | C17H14O5 | 297.2429 | 0.8863 | M-H |
| P82 | 15.5123 | Astragaloside IV | C41H68O14 | 783.4518 | 0.9251 | M-H |
| P83 | 15.5283 | Majonoside R2 | C41H70O14 | 785.4678 | 0.5515 | M-H |
| P84 | 16.1173 | Vinaginsenoside R2 | C43H72O15 | 827.4797 | 0.6976 | M-H |
| P85 | 16.3507 | Dioscin | C45H72O16 | 867.4739 | 0.7983 | M-H |
| P86 | 16.6069 | Capsicoside C2 | C44H72O17 | 871.4697 | 0.6151 | M-H |
| P87 | 17.1165 | α-Irone | C14H22O | 205.1589 | 0.8650 | M-H |
| P88 | 17.3491 | Schidigerasaponin D1 | C44H72O17 | 871.4701 | 0.6715 | M-H |
| P89 | 17.8673 | Cholesteryl laurate | C39H68O2 | 283.2642 | 0.9385 | M-2H |
