# Supplementary figures and images for: Jianpi Yangzheng Xiaozheng decoction alleviates gastric cancer progression via suppressing exosomal PD-L1
Source: Front Pharmacol. 2023 Aug 3;14:1159829. doi: 10.3389/fphar.2023.1159829 (PMC10434994; doi:10.3389/fphar.2023.1159829)

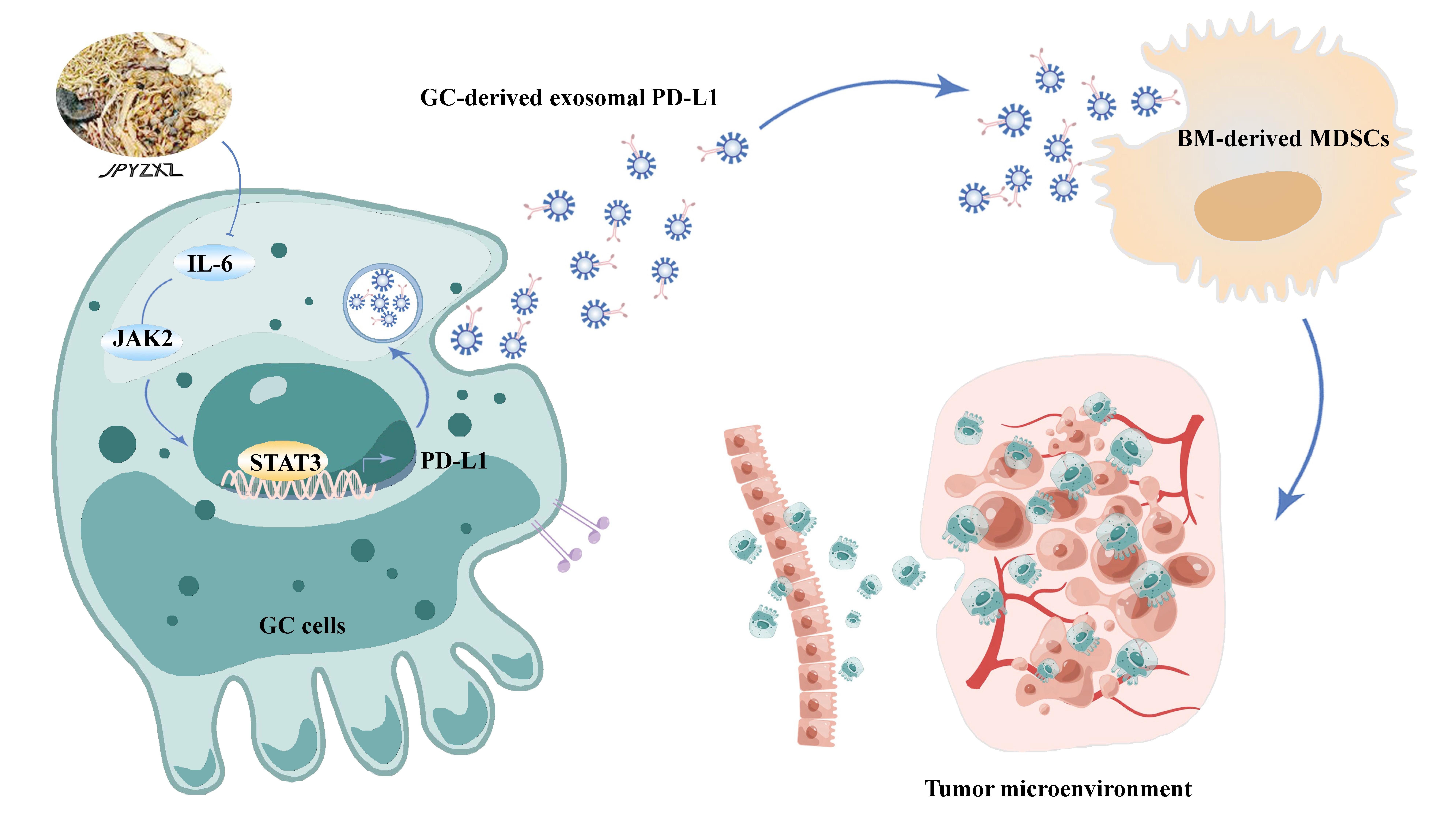

Supplement: Supplementary file 2 [file Image1.JPEG]

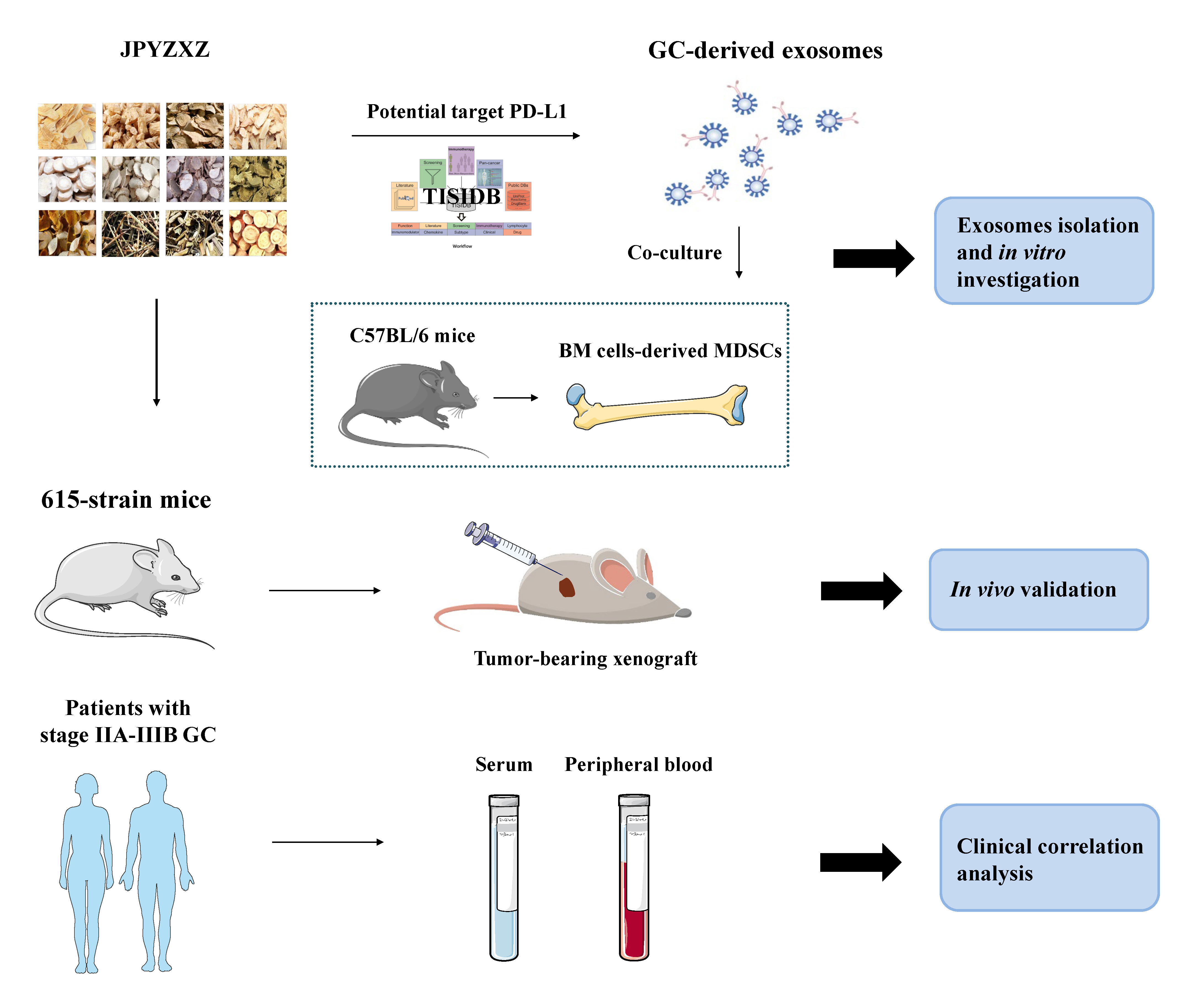

Supplement: Supplementary file 3 [file Image2.JPEG]
